# Precise programming of multigene expression stoichiometry in mammalian cells by a modular and programmable transcriptional system

**DOI:** 10.1038/s41467-023-37244-y

**Published:** 2023-03-17

**Authors:** Chenrui Qin, Yanhui Xiang, Jie Liu, Ruilin Zhang, Ziming Liu, Tingting Li, Zhi Sun, Xiaoyi Ouyang, Yeqing Zong, Haoqian M. Zhang, Qi Ouyang, Long Qian, Chunbo Lou

**Affiliations:** 1 Center for Quantitative Biology, Academy for Advanced Interdisciplinary Studies, Peking University, 100871 Beijing, China; 2 Peking-Tsinghua Joint Center for Life Sciences, Peking University, 100871 Beijing, China; 3 Center for Cell and Gene Circuit Design, CAS Key Laboratory of Quantitative Engineering Biology, Guangdong Provincial Key Laboratory of Synthetic Genomics, Shenzhen Key Laboratory of Synthetic Genomics, Shenzhen Institute of Synthetic Biology, Shenzhen Institutes of Advanced Technology, Chinese Academy of Sciences, 518055 Shenzhen, China; 4 Yuanpei College, Peking University, 100871 Beijing, China; 5 College of Life Science, University of Chinese Academy of Science, 100149 Beijing, China; 6 Bluepha Co., Ltd, 102206 Beijing, China

**Keywords:** Synthetic biology, Synthetic biology

## Abstract

Context-dependency of mammalian transcriptional elements has hindered the quantitative investigation of multigene expression stoichiometry and its biological functions. Here, we describe a host- and local DNA context-independent transcription system to gradually fine-tune single and multiple gene expression with predictable stoichiometries. The mammalian transcription system is composed of a library of modular and programmable promoters from bacteriophage and its cognate RNA polymerase (RNAP) fused to a capping enzyme. The relative expression of single genes is quantitatively determined by the relative binding affinity of the RNAP to the promoters, while multigene expression stoichiometry is predicted by a simple biochemical model with resource competition. We use these programmable and modular promoters to predictably tune the expression of three components of an influenza A virus-like particle (VLP). Optimized stoichiometry leads to a 2-fold yield of intact VLP complexes. The host-independent orthogonal transcription system provides a platform for dose-dependent control of multiple protein expression which may be applied for advanced vaccine engineering, cell-fate programming and other therapeutic applications.

## Abstract (web summary)

Context-dependency of mammalian transcriptional elements has hindered the quantitative investigation of multigene expression stoichiometry and its biological functions. Here the authors present a host-orthogonal transcriptional system that drives tunable gene expression in mammalian cells, enabling predictive fine-tuning of multi-gene expression stoichiometry and the production optimization of virus-like particles from mammalian cells.

## Introduction

Optimal multigene expression stoichiometry is essential for maintaining protein homeostasis of protein-complex subunits or pathway-specific enzymes in almost all investigated organisms from bacteria to the eukaryotic cells^1–5^. In higher eukaryotes, disruptive changes in protein dosages by gains or losses of the chromosomes could trigger aberrant aggregations of the proteins in excess^6^, and result in pathological cellular states such as tumor and neurodegeneration^7^. To maintain the desired protein stoichiometry and dosage, cells employ several regulatory mechanisms to buffer protein levels against environmental and genetic fluctuations, including RNA-based regulation, diffusion-limited mRNA nuclear export, and targeted non-exponential degradation of unassembled subunits of multiprotein complexes^8,9^. Specifically, it was unveiled that the stoichiometry of obligate subunits of multiprotein complexes was proportionally controlled by their synthesis rates rather than post-translational negative feedback in human cells and other eukaryotic cells^3^.

From an engineering perspective, the importance of an appropriate stoichiometry in mammalian multigene expression has only recently been fully acknowledged by those in fundamental and therapeutic research such as stem-cell fate programming, multiprotein vaccine production, and GPCR platform reconstitution for drug screening^10^. For example, the stoichiometry of four reprogramming factors (Oct4, Sox2, Klf4 and c-Myc) dramatically affected the efficiency and quality of induced pluripotent stem cells^11,12^. However, translational control of protein dosage in mammalian cells is challenging, as methods such as introduction of internal ribosome entry sites or rank shuffling of co-translated proteins have limited tunability. Alternatively, on the transcriptional level, a handful of well-characterized mammalian promoters enable dosage control of the desired proteins. However, their activities are sensitive to their genetic context and thereby do not suffice for the precise fine-tuning of stoichiometry^13,14^. On the contrary, insulated and composable promoters have been designed with their activities predicted by biochemical models in bacteria. But such promoters remain underexplored in mammalian cells^15,16^. Specifically, the transferability of these context-independent regulatory elements is challenged by the complex transcriptional pipeline in mammalian cells.

Here, we present a strategy to program the stoichiometry and dosage of multi-protein expression in mammalian cells. A library of host-independent and modular promoters derived from bacteriophage with predetermined binding affinities was introduced into mammalian cells, and specifically transcribed by a monomeric RNA polymerase (RNAP) fused with an mRNA capping enzyme. We showed that by introducing this exogenous transcriptional apparatus, synthetic transcriptional regulation became insulated from the host cell which allowed clear mechanistic modeling of multigene expression control. In particular, a biochemical model involving multiple transcriptional units was developed based on single promoter models and predetermined model parameters. By introducing a resource-competition mechanism, the model was used to quantitatively predict the expression stoichiometry of multiple genes. Finally, we applied the model-guided stoichiometric programming strategy to the production optimization of virus-like particles (VLPs) for a highly pathogenic subtype H1N1 of the influenza A virus. We expect the strategy to facilitate developments in advanced vaccine engineering, stem cell engineering, and other biomedical applications.

## Results

### A host-independent and programmable expression system enabled predictive control of transgene expression in mammalian cells

**Fig. 1:**
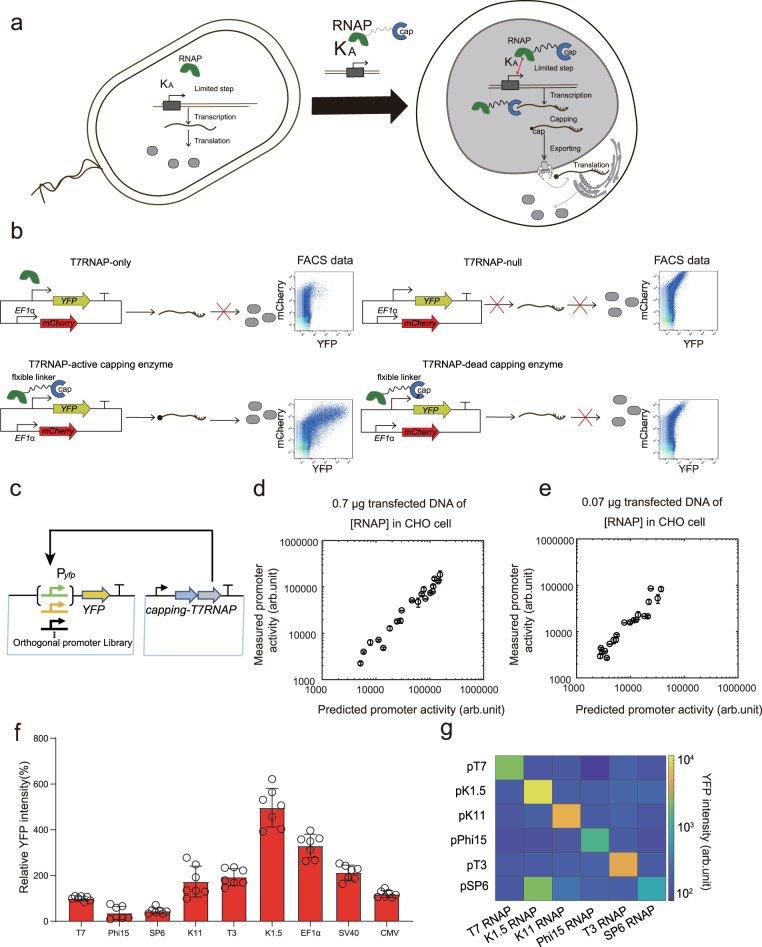
Design and characterization of the orthogonal transcription system. **a** The binding of the orthogonal RNAP to its cognate promoters (with binding affinity *K*_A_) is a limiting step for protein synthesis in all organisms. The capping enzyme fused to RNAP ensures nuclear export and translation efficiency of the synthetic transcripts in mammalian cells. **b** The reporter gene expressions for the T7 promoter with RNAP-null, RNAP-only, RNAP-dead capping enzyme and RNAP-active capping enzyme. **c** Scheme of the quantitative measurement circuit composed of a monomeric RNA polymerase (RNAP) fused to a capping enzyme driven by the EF1α promoter and a set of T7 promoter variants in two separated plasmids. **d** Linearity between expression measured by flow cytometry and expression predicted by model in CHO cells transfected with 0.7 μg T7 RNAP plasmid. **e** Linearity between expression measured by flow cytometry and expression predicted by model in CHO cells transfected with 0.07 μg T7 RNAP plasmid. Data represent the mean ± SD (*n* = 3) **f** Comparison of expression from six different orthogonal promoters driven by cognate RNAPs and three widely-used mammalian promoters (CMV, EF1α and SV40) driven by the endogenous RNAP. Data represent the mean ± SD (*n* ≥ 6). **g** Characterization of a 6 × 6 orthogonality matrix of host-independent RNAPs and their cognate promoters. All data represent the mean ± SD (*n* ≥ 3). Source data are provided as a Source data file.

Compared with simple prokaryotic cells, protein expression in mammalian cells is subject to multiple layers of regulation, including pre-mRNA splicing, mRNA capping modification, poly-A addition, the export of mRNA from the nucleus to the cytoplasm, and so on. To overcome these barriers, we introduced an RNAP from T7 bacteriophage fused with an mRNA capping enzyme of viral origin via a long flexible linker (GGGGSGGGGSGGGGSGGGGS)^17^. We speculated this orthogonal apparatus and its cognate promoters would shield transgene expression from host interferences and serve as a basis for fine-tuning protein expression stoichiometry in mammalian cells (Fig. 1a). As the relative binding affinities for more than 50 modular T7 promoters were quantitatively determined in our previous study^15^, we tested the above hypothesis in mammalian cells by interrogating the relationship between the transcriptional activity of the programmable and modular promoters and their binding affinity to the cognate RNAP.

To construct a quantitative measurement system, we inserted individuals of 20 chosen promoters upstream of a reporter gene (*yfp*) in one plasmid, and another plasmid carried a constitutively expressed gene encoding the fusion protein of RNAP and capping enzyme (Fig. 1b, c). The two plasmids were co-transfected into the Chinese hamster ovary (CHO) cell line or the human embryonic kidney 293 T (HEK293T) cell line. The promoter activities were quantified by flow cytometry. A constitutively expressed red fluorescent protein (mCherry) was used to indicate the plasmid-transfected cell population. Surprisingly, we found that the activities of the host-independent and modular promoters held a positive correlation within 100-fold variation with their pre-determined binding affinities when the cognate RNAP was expressed at a proper level (Supplementary Fig. 3a), and fusion of the capping enzyme was necessary for translation (Fig. 1b). We thus built a simple biochemical model to predict the activity of single promoters driven by the RNAP fusion protein (referred to as RNAP thereinafter) (Eq. 1).

1 $$\left[P\right]=\left(\alpha {\left(\frac{\left[{{{{{{\rm{RNAP}}}}}}}\right]}{{K}_{{{{{{\rm{D}}}}}}}}\right)}^{n}\right)\Big/\left(1+{\left(\frac{\left[{{{{{{\rm{RNAP}}}}}}}\right]}{{K}_{{{{{{\rm{D}}}}}}}}\right)}^{n}\right)+\beta$$

where *α* is the maximal expression for the promoters with enough cognate RNAP, *β* is the basal expression of the promoters without RNAP, *K*_A_ = 1/*K*_D_ is the relative binding affinity of the promoters to the cognate RNAP, *n* is the cooperative factor of RNAP accessing the promoter, and $[{{{{{{\rm{RNAP}}}}}}}]$ and $\left[P\right]$ are the concentrations of the RNAP and the reporter protein, respectively. These parameters were fitted from the data of Supplementary Fig. 3, which were robust to parameter-perturbations and slightly different in CHO and HEK293T cells (Supplementary Table 1).

For the 20 selected promoter sequences in CHO cells, the predicted promoter activities had a linear relationship with the measured promoter activities within the 100-fold activity range (Fig. 1d). The sequences of the 20 promoters were listed in Table S4. The linear relationship persisted when we tuned down the RNAP concentration by reducing 10-fold the amount of transfected RNAP plasmids (Fig. 1e), though in this experiment, some of the constructs had reached the minimal expression levels (Supplementary Fig. S3a). For the strongest T7 promoter, we gradually changed the plasmid concentration of the fused RNAP-capping gene and found that the promoter activities were dependent on the RNAP concentration, which was consistent with the above biochemical model (Supplementary Fig. 1). The biochemical model held its predictive power when we shifted the host to HEK293T cells (Supplementary Fig. 3c, d). Furthermore, five other RNAPs from bacteriophages were fused with the same capping enzyme and had their activities tested. One of the fusion proteins elicited stronger reporter gene expression than the T7RNAP fusion protein and several widely used constitutive promoters (Fig. 1f). We demonstrated that the six RNAPs could orthogonally recognize their cognate promoters, suggesting the potential of expanding the complexity of the host-independent mammalian regulatory system (Fig. 1g). In the following experiments to characterize the host-independent expression system, we used the T7 RNAP as a representative unless stated otherwise.

### Resource competition played a key role in multigene expression stoichiometry

**Fig. 2:**
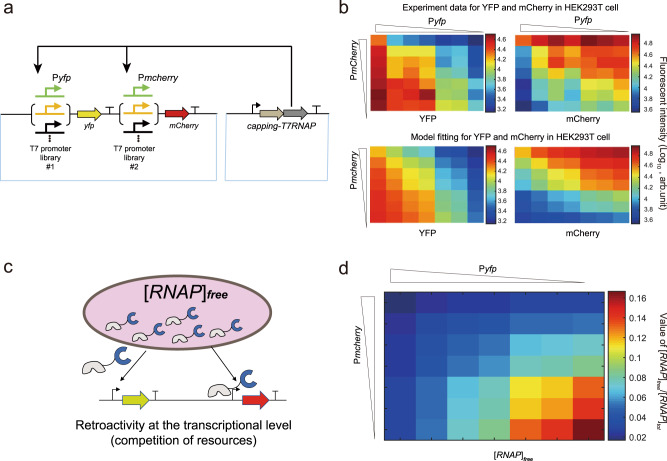
Resource competition model in the two-reporter system. **a** The genetic circuit design for the RNAP-driven expression of two reporter genes. **b** Characterization of resource competition by two co-expressed reporters in HEK293T. Experimental data and model predictions are compared at varying activities for the two reporters. Data represent mean values of more than three biological replicates. **c** Schematic of different promoters competing for the same pool of free RNAP ([RNAP]_free_). **d** Heatmap showing the concentration of [RNAP]_free_ as calculated by the biochemical model with resource competition. Promoter activities are decreased as shown by the triangles for two reporter genes. Source data are provided as a Source data file.

The cellular and genetic contexts are two factors possibly affecting the stoichiometry of multigene expression in mammalian cells^13,18,19^. One well-studied context effect is caused by the competition of key resources among the expressed genes. To test its effect in our system, we designed a two-reporter (*yfp*, *mCherry*) system in mammalian cell lines (Fig. 2a). We first confirmed the linear relationships between the fluorescence data and the transcribed mRNA level by quantitative-PCR methods (Supplementary Fig. 7). In the two-reporter system, each reporter gene was controlled by one of seven representative promoters from the T7 promoter library (Supplementary Table 5), resulting in 49 different combinations. We found that a strong promoter for one gene dramatically decreased the expression of another gene no matter how strong the other gene’s promoter was (Supplementary Figs. 2b and 5). This result indicated that the two reporter genes competed for some common resources. The same resource competition phenomenon was observed when the *mCherry* gene was replaced by a *bfp* gene (Supplementary Fig. 6). We speculated the limited resource for promoter competition was the available RNAP not engaged in transcription. To further test the resource-limiting model, we designed a two-reporter (*yfp*, *mCherry*) system driven by two orthogonal pT7 and pSP6 promoters which were transcribed by T7 and SP6 RNAP, respectively. The reporter genes were downstream of T7-cognate or SP6-cognate promoters with high, medium, or low activity, yielding 3 × 3 = 9 dual reporter constructions. We found that the activities of SP6 promoters varied mostly independently with the activities of T7 promoter strengths, while the activities of T7 promoters slightly increased with a decreasing SP6 promoter strength. These results supported that the two orthogonal promoters did not compete the key resource-limiting factors (Supplementary Fig. 8).

To integrate the resource-competition mechanism, we expanded the simple model by replacing the total RNAP concentration (${\left[{{{{{{\rm{RNAP}}}}}}}\right]}_{{{{{{{\rm{tot}}}}}}}}$) with the free RNAP concentration $({[{{{{{{\rm{RNAP}}}}}}}]}_{{{{{{{\rm{free}}}}}}}})$ (Eqs. 2–6 and Fig. 2c).

2 $$\left[{{{{{{\rm{YFP}}}}}}}\right]=\left({\alpha }_{{{{{{{\rm{yfp}}}}}}}}[{{{{{\rm{DNA}}}}}}]_{{{{{{{\rm{tot}}}}}}}}{\left(\frac{{\left[{{{{{{\rm{RNAP}}}}}}}\right]}_{{{{{{{\rm{free}}}}}}}}}{{K}_{{{{{{{\rm{yfp}}}}}}}}}\right)}^{n}\right)\Big/\left(1+{\left(\frac{{[{{{{{{\rm{RNAP}}}}}}}]}_{{{{{{{\rm{free}}}}}}}}}{{K}_{{{{{{{\rm{yfp}}}}}}}}}\right)}^{n}\right)+{\beta }_{{{{{{{\rm{yfp}}}}}}}}$$

3 $$\left[{{{{{{\rm{mCherry}}}}}}}\right]=\left({\alpha }_{{{{{{{\rm{mCherry}}}}}}}}[{{{{{\rm{DNA}}}}}}]_{{{{{{{\rm{tot}}}}}}}}{\left(\frac{{\left[{{{{{{\rm{RNAP}}}}}}}\right]}_{{{{{{{\rm{free}}}}}}}}}{{K}_{{{{{{{\rm{mCherry}}}}}}}}}\right)}^{n}\right)\Big/\left(1+{\left(\frac{{\left[{{{{{{\rm{RNAP}}}}}}}\right]}_{{{{{{{\rm{free}}}}}}}}}{{K}_{{{{{{{\rm{mCherry}}}}}}}}}\right)}^{n}\right)+{\beta }_{{{{{{{\rm{mCherry}}}}}}}}$$

4 $${[{{{{{{\rm{RNAP}}}}}}}]}_{{{{{{{\rm{free}}}}}}}}={[{{{{{{\rm{RNAP}}}}}}}]}_{{{{{{{\rm{tot}}}}}}}}/\left(1+[{{{{{\rm{DNA}}}}}}]_{{{{{{{\rm{yfp}}}}}}}\;{{{{{{\rm{free}}}}}}}}/{K}_{{{{{{{\rm{yfp}}}}}}}}+[{{{{{\rm{DNA}}}}}}]_{{{{{{\rm{mCherry}}}}}}\;{{{{{{\rm{free}}}}}}}}/{K}_{{{{{{{\rm{mCherry}}}}}}}}\right)$$

5 $$[{{{{{\rm{DNA}}}}}}]_{{{{{{{\rm{yfp}}}}}}}\;{{{{{{\rm{free}}}}}}}}=[{{{{{\rm{DNA}}}}}}]_{{{{{{{\rm{tot}}}}}}}}\Big/\left(1+{\left(\frac{{\left[{{{{{{\rm{RNAP}}}}}}}\right]}_{{{{{{{\rm{free}}}}}}}}}{{K}_{{{{{{{\rm{yfp}}}}}}}}}\right)}^{n}\right)$$

6 $$[{{{{{\rm{DNA}}}}}}]_{{{{{{{\rm{mCherry}}}}}}}\;{{{{{{\rm{free}}}}}}}}=[{{{{{\rm{DNA}}}}}}]_{{{{{{{\rm{tot}}}}}}}}\Big/\left(1+{\left(\frac{{\left[{{{{{{\rm{RNAP}}}}}}}\right]}_{{{{{{{\rm{free}}}}}}}}}{{K}_{{{{{{{\rm{mCherry}}}}}}}}}\right)}^{n}\right)$$

All variables and parameters are the same as in Eq. 1 and gene-specific parameters were indexed. The additional variable [DNA]_tot_ represented the concentration of the transfected plasmids. After acquiring the new parameters by fitting experimental data from 49 combinations of dual promoters driving two reporter genes, we found a nonuniform distribution for the free RNAP concentration ([RNAP]_free_). The concentration was the lowest when both promoters were the strongest, became higher when either promoter was weakened and was the highest when both promoters were the weakest (Fig. 2d).

**Fig. 3:**
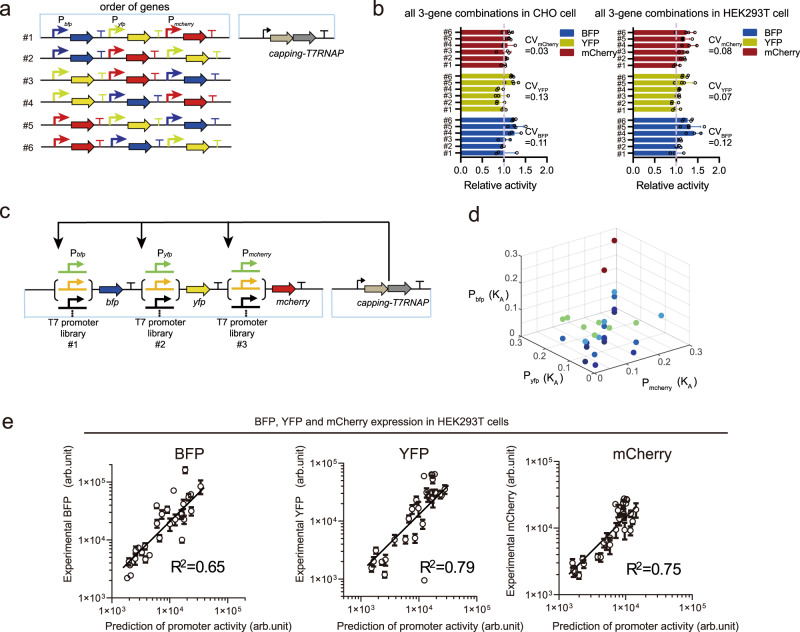
Gene-order effect for three-gene expression and predictions of the resource-competition model. **a** Schematic of the three-reporter characterization system with all possible orders of the transcriptional units. **b** Relative activity of blue, yellow and red fluorescent proteins of each reporter gene (*bfp*, *yfp*, and *mCherry*) based on their respective expression level in construct #1. Data represent the mean ± SD (*n* = 4). **c** Schematic of the circuit for the performance evaluation of three-reporter gene expression system. **d** Three-dimensional graph of the combinatorial promoter library with different binding affinities to the cognate RNAP. Different colors show different *z*-axis (*P*_*bfp*_ value). **e** Correlation between predicted promoter activities and experimental fluorescent intensities on a log–log scale for the three reporter genes. Data represent the mean ± SD (*n* = 3). Source data are provided as a Source data file.

For transiently transfected expression systems, the second factor affecting expression stoichiometry is the genetic context, which includes the local DNA sequence and the gene order^14,20,21^. We thus performed a full shuffling of the order of three reporter genes (*yfp*, *mCherry*, and *bfp*) and quantified the variance in each reporter’s expression in all six combinations (Fig. 3a). The calculated coefficient of variations (CV) among expressions from one promoter in all orders were very small (CV_YFP_ = 0.07, CV_mCherry_ = 0.08, CV_BFP_ = 0.12 in HEK293T cells; CV_YFP_ = 0.13, CV_mCherry_ = 0.03, CV_BFP_ = 0.11 in CHO cells, Fig. 3b). This result indicated that the promoter activities were not substantially affected by their local genetic contexts, i.e., these modular promoters enabled flexible and robust multigene expression tuning despite of the local DNA sequence and the gene order.

With a fixed order of three reporter genes (*bfp-yfp*-*mCherry*), we chose several combinations for their promoters within the 100-fold spectrum of binding affinities and evaluated the programmability of their expression stoichiometry by the resource competition model (Fig. 3c, d). With all parameters fitted from the two-gene systems (Fig. 2b), we found the protein expressions for all three genes in both mammalian cell lines were predicted by the model with Pearson correlation coefficients from 0.81 to 0.88 between model predictions and experimental measurements (Fig. 3e and Supplementary Fig. 10). Taken together, the host-independent transcription system we employed was insensitive to the genetic context and enabled predictable design of multigene expression stoichiometry through the resource competition for their cognate RNAP.

### Production of H1N1 VLPs was optimized by stoichiometry programming of three-component proteins

**Fig. 4:**
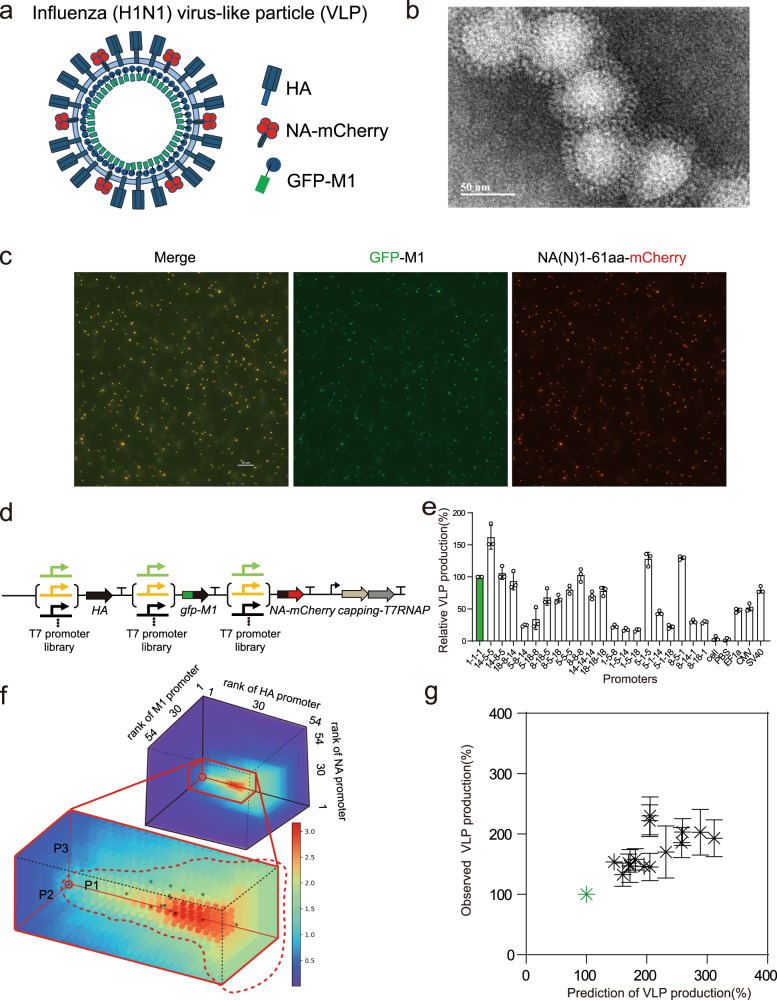
Stoichiometry optimization of the three components for the influenza A subtype H1N1 VLP via the predictive model. **a** Schematic of the recombinant VLP in which NA and M1 proteins are fused with mCherry and GFP reporter proteins, respectively. **b** Transmission electron microscopy images of influenza VLPs. Scale bar, 50 nm. **c** Representative images of the VLPs produced from the three-gene system. Fluorescent microscopy was used to observe co-localization events of NA and M1. Scale bar, 10 μm. **d** Schematic of the plasmid constructions for VLP production. **e** Relative VLP yields for 21 selected promoter-gene combinations and 3 widely-used mammalian promoters (EF1α, CMV and SV40). The reference VLP yield was of the combination of the strongest promoters and was assigned as 100%. Data represent the mean ± SD (*n* = 3). **f** Predicted VLP yields in all 157,464 combinations of the available HA, M1 and NA promoters. The reference combination is normalized to 1. The dashed circle marks the rough boundary of the promoter combinations with yields higher than that of the reference. Stars indicate the combinations selected for experimental validation. **g** Comparison of experimental VLP yields with model predictions. Data represent the mean ± SD (*n* = 3). Source data are provided as a Source data file.

Virus-like particles (VLPs) are proteinaceous nanoparticles that are nonpathogenic and incapable of replication. Synthetic VLPs have broad applicational potential in biomedical areas such as vaccines and drug delivery. The stoichiometry of protein subunits is critical for VLP’s assembly efficiency and immunogenicity^22,23^. We therefore investigated the programmability of stoichiometry and its impact on VLP yield. We first verified that with proper stoichiometry, three key subunits (hemagglutinin (HA), neuraminidase (NA), and matrix protein 1 (M1)) of the influenza A virus could form stable VLPs (Figs. 4a and 4b). Following previous studies, we constructed two fusion genes, *egfp*-*M1* and *NA*-*mCherry*, to monitor the production of intact VLPs by fluorescent microscopy or flow cytometry (Fig. 4a). Most M1-containing (green) VLPs co-localized with NA-containing (red) ones, whereas some NA-containing VLPs did not contain M1 (Fig. 4c and Supplementary Fig. 11). We thus chose green fluorescence as the indicator to quantify the yield of the influenza A VLPs by flow cytometry. Next, we chose the three genes from the highly pathogenic influenza A virus subtype H1N1 to demonstrate the programmability of the host-independent transcriptional system. To vary the stoichiometry of the 3 subunits, 21 combinations of different promoters were chosen for multigene expression control (Fig. 4d). We found that the strongest promoters did not elicit the highest yield of VLPs while weaker promoters for certain subunits produced more VLPs (Fig. 4e).

The above resource competition model was employed to predict the yield of VLPs with predetermined parameters and gene-specific parameters (Table S3). In silico screen of all of the 54 × 54 × 54 = 157,464 promoter combinations suggested that a high expression of HA protein was harmful to the production of intact VLPs, and proper stoichiometric combinations of the three genes could produce twofold more than the combination of the strongest expressions in each gene alone (red circle in Fig. 4f, referred to as the “reference” hereinafter). From the region in the promoter space where predicted VLP production was higher than that of the reference, we chose some of the high-yield combinations for experimental validation. All chosen combinations produced more VLPs than the reference, and their experimental yields were substantially predicted by the resource-competition model without any free parameter (Fig. 4g). In conclusion, based on the host-independent, modular and programmable transcription system, the multi-protein stoichiometry for an important VLP complex was optimized, as guided by a biochemical model in a predictable and finely tunable manner.

## Discussion

In this work, we developed an approach to precisely titrate the stoichiometry of multigene expression in mammalian cells using a host-independent and programmable transcriptional system. The promoter activities of single and multiple gene expression could be uniformly varied and their expression outputs were predictable by minimal biochemical models that revealed mechanistic insights. As an example, the stoichiometry of three genes of an influenza A virus VLP was systematically screened and optimized to attain a high yield of intact particles. These results lend support to the utility of the host-independent regulatory elements in the predictive design of complex synthetic circuits in mammalian cells for advanced quantitative bioengineering in basic research and biomedical applications.

For engineered transcriptional regulatory systems that rely on the host transcriptional machineries, the activities of either synthetic or natural promoters are affected by their gene order, genomic loci, epigenetic modification, and host cell types, and it is often difficult to precisely control the stoichiometry of multiple genes^13,24,25^. While certain natural genes and pathways have evolved to be stoichiometric through concerted changes in a combination of regulatory elements (mostly in *cis*), synthetic constructions of stoichiometric expressions are often ruined by those of the above elements that are outside the design realm. First, the gene order of multiple genes was reported to have substantially affected their expression from their respective promoters. Specifically, the expression of downstream genes was often reduced by the upstream gene^13^. Second, widely used constitutive promoters of mammalian or viral origins displayed cell type-specific gene expression patterns and unpredictable silencing. For example, in seven cell lines including HEK293T, the relative gene expressions from the human elongation factor 1α (EF1α) promoter and other mammalian promoters were dramatically changed across cell types^26^. In primary motor neuron cells, the strong cytomegalovirus (CMV) promoter drove a high initial expression but its activity gradually diminished afterwards^27^. On the contrary, the relative activities of our modular promoters are independent of their gene orders. Rank shuffling of triplet genes did not significantly affect their individual expression levels. We also showed the system’s consistency in two mammalian cell lines as well as in several prokaryotes. It is of great interest to further test the system’s sensitivity to other genetic contexts and host factors.

The predictive models employed are simple biochemical models with basic parameters to describe the minimal and maximal expression activities (*α*, *β*), the Hill coefficient (*n*) and the half-maximal binding affinity (*K*). We performed parameter sensitivity analyses to find that all fitted parameters were robust to perturbations. In some cases (e.g., Fig. S3), the non-linearities observed between model predictions and experimental measurements were possibly due to over-estimated minimal expression and under-estimated binding affinities. Data for a broader RNAP expression range will be needed to fix these biases and further increase the precision of model prediction.

Systematic investigations of the crucial role of multigene expression stoichiometry on biological functions and evolutionary fitness have been hindered by the lack of tools to titrate their expression in a dose-dependent manner. Several recent studies have highlighted this critical issue and proposed CRISPR-Cas9-based technology^25,28^. This high throughput approach is suitable for the large-scale evaluation of the phenotypic effects of single genes. Adapting such perturbation approaches to quantify the phenotypic effects of multi-gene stoichiometry requires careful modeling of the competition effects over relevant resources such as cellular RNAP and dCas9 proteins. By supplementing the multigene expression vector with its own transcriptional and mRNA capping machineries, we were able to decouple the circuit from host transcriptional machineries and thereby reduce the well-studied transcriptional level interference^29,30^ from host contexts, as evidenced by the predictive power of our simple biochemical models on the multigene expression stoichiometry. One possible challenge for the host-independent system is that it may take too much translational resources and induce unexpected cellular burden or toxicity for mammalian cells. Other challenges would be the resource-limiting factors coming from translational process and other regulatory processes^31–33^. For example, ribosomes and other translational factors were found to be the limiting resources for expressing multiple genes^31^. Dicer proteins as a common cofactor of miRNA regulations would be a limiting factor for the regulatory functions of multiple miRNAs, which could result in retroactivity for genetic circuits composed of multiple miRNAs^32^.

In summary, a host-independent and programmable transcriptional platform in mammalian cell was demonstrated to gradually and precisely control the expression dosage of single genes and the expression stoichiometry of multiple genes. The precise control of stoichiometry will empower us to program the differentiation and trans-differentiation of stem cells, as well as the orchestrated expression of multiprotein for applications in advanced vaccines.

## Methods

### Cell culture

Chinese Hamster Ovary cells (CHO, National Infrastructure Cell Line Resource, NICR), Capping-T7-RNAP stable cell line (B9), and Human Embryonic Kidney 293 T cells (HEK293T, ATCC) were cultivated in High Glucose Dulbecco’s modified Eagle’s media (DMEM-high glucose, Hyclone), supplemented with 10% fetal bovine serum (FBS, GIBCO) and 1% penicillin–streptomycin (Hyclone). All cell cultures were kept under 5% CO_2_ and 37 °C temperature.

### Plasmid construction

Plasmids used in this study were constructed from two basic vectors, pOMC1 and pOMC2, which were modified versions of the PiggyBac system. Plasmid maps can be found at http://bdainformatics.org/dataRepository. The coding sequences of orthogonal transcription system were synthesized (Genewiz) and optimized for expression in human cells. Basic parts (promoters, 5’UTRs, coding sequences, 3’UTRs, and terminators) were created via standard cloning techniques. For constructing Capping-RNAP, backbone vectors were linearized by restriction endonucleases (New England Biolabs, NEB), and the insert DNA fragments (the ORFs of Capping-RNAP) were PCR amplified from plasmid using PrimeSTAR Max DNA Polymerase (TAKARA). pOMC1 with a truncated hEF1α-driven transfection marker was used to construct the cassettes for the “programmable and modular promoter”-driving reporters. The multi-reporter systems or VLP-producing plasmids were constructed using a Golden Gate strategy. Transcription units for expressing fluorescent reporters: YFP (citrine), RFP (mCherry), BFP (tagBFP) or the VLP-formation proteins: Hemagglutinin (HA/Brisbane, H1N1), Neuraminidase (NA/WSN, H1N1), matrix protein 1 (M1, H1N1), GFP-M1 and NA(1-61aa)-mCherry were amplified by PCR and assembled into multi-TU plasmids using SapI (NEB) Golden Gate reactions. Assembly primers and sequences were provided in Supplementary Table S10. Plasmids were transformed into Top10 or DH5α *E.coil* competent cells and these cells were plated on LB agar and propagated in LB media. All plasmids were extracted from cells with QIAprep Spin Miniprep.

### Cell transfections

All plasmids were transfected into cells by Lipofectamine 3000 (Invitrogen) according to the manufacturer’s instructions. Typically, 1.5–2.0 × 10^5^ cells were seeded on 24-well plates, and upon transfection, the confluency of cells was around 70–80%. For testing of single-gene reporter library, 0.3 μg of each T7 promoter library plasmid with a reporter was co-transfected with 0.7 or 0.07 μg of Capping-T7 RNAP plasmid in 24-well plates. For RNAP orthogonality analysis, 0.5 μg of RNAP plasmids and 0.5 μg of reporter plasmid were co-transfected in 24 well plates. For libraries of two/three-reporters or positional effect analysis, 0.5 μg of each plasmid was transfected in 24 well plates. For virus-like particles (VLP) purified by the sucrose cushion, 50 μg of each plasmid was transfected into CHO cells in 15 cm dishes. For VLP quantified by Apogee Micro Flow Cytometer, 2 μg of each VLP production plasmid was transfected into B9 cells harboring an existing T7 Capping-RNAP integration in 6 well plates.

### Flow cytometry and real-time PCR methods

Preparing samples in 96-well plates for flow cytometry. For transfection experiments, cells were transfected 2d before being harvested. Then, media was aspired, 100 μl PBS was added to wash the cells, the PBS was aspired, and 40 μl Trypsin-EDTA (GIBCO) was added. Following this, cells were suspended and transferred to 96-well plates. The plated were spun down at 200 × *g*, 5 min, and the media was aspirated. Cells were resuspended in 200 μl 4% paraformaldehyde (PFA, Boster Biological Technology). One hundred thousand cells were analyzed for each sample on a Beckman Coulter CytoFlex S flow cytometry or BD Fortessa SORP equipped with proper lasers and filters, and data were analyzed with CytoExpert (Beckman Coulter) or FlowJo (V10) software, respectively. Some of the flow cytometry data of Figs. 1–4 are given in Supplementary Materials (Supplementary Figs. 2, 4, and 9). Real-time qPCR was performed by RuiBiotech Co., Ltd. The primers used to quantify Citrine mRNA levels and CHO GAPDH (*Cricetulus griseus*) primers used for normalization were as follows:

GAPDH-CHO-F: 5’-GAAGGTGGTGAAGCAGGCAT;

GAPDH-CHO-R: 5’-CAGCCCCAGCATCAAAGGTG;

Citrine-F: 5’-TCAAGGACGACGGCAACTAC;

Citrine-R: 5’-GTCCTCCTTGAAGTCGATGC.

### Construction of capping-T7 RNAP stable cell lines

A stable cell line was generated with piggyBac transposon system as described by the user manual. More specifically, 0.75 μg of capping-T7 RNAP plasmid was co-transfected with 0.25 μg of transposon plasmid in CHO cells cultivated in a 6-well plate. Forty-eight hours post-transfection by Lipofectamine 3000, cells were split into three 48-well plates. When the confluency reached 30–50%, 400 μg/ml of hygromycin was added to select the positive clone. After antibiotic selection for 1–2 weeks, the positive clones were verified through T7 reporter expression level by flow cytometry (Beckman, CytoFlex S). B9 clone was selected for subsequent experiments.

### The VLP purification

VLP samples were purified by sucrose cushion method modified from Buffin^34^. Briefly, cell culture media were collected at 72 h after transfection and clarified sequentially by centrifugation at 300 × *g*, 5 min, and 3000 × *g*, 10 min. After filtrated with a 0.45-μm filter (Millipore), the supernatant was loaded above the 30% w/v sucrose solution (dissolved with phosphate-buffered saline (PBS)), and centrifugated by ultracentrifugation with a P32ST (Himac) swinging bucket rotor at 150,000 × *g*, 4 °C for 3 h. The VLP pellets were resuspended in 100 μl of PBS.

### Transmission electron microscope (TEM)

Ten microliters of VLP samples purified by sucrose cushion were incubated on carbon-coated copper grids (Beijing Zhongjingkeyi Technology) for 10 min at room temperature (RT). The excess samples were carefully sucked out of the grid with filter paper. Then the samples on the grids were stained with 2% phosphotungstic acid for 1 min at RT. Excess stain was drained off with filter paper. Grids were dried overnight and scanned by Transmission Electron Microscope (FEI Tecnai G2 F20).

### Microscope images of fluorescent VLP and colocalization analysis

The microscope protocol was modified from Gonzalez-Dominguez^35^. Briefly, 1.5 μl of harvested fluorescent VLP samples were loaded on the clean microscope slides and carefully spread by cover glass (diameter = 15 mm). Green and Red channel images were captured on the Nikon Ti2-E microscope with a ×100 oil objective and an ANDOR (ZYLA) camera under the control of NIS-elements software (Nikon). Colocalization of green and red signals was analyzed with the spot analysis program in Imaris software (Oxford Instruments, supported by Yilin Wang from Southern University of Science and Technology).

### Fluorescent VLP quantified by Apogee Micro Flow Cytometer

Samples were prepared as mentioned in VLP purification except omitting the sucrose clarifying step and analyzed by Apogee Micro Flow Cytometer (Apogee Flow Systems) with the 488 nm laser using its extracellular vesicles program. Data were analyzed with Histogram software (Apogee Flow Systems).

### Modeling and prediction

For two reporters of YFP-BFP, mCherry could be substituted with BFP in Eqs. 2–6; for prediction of three reporters, equations were similar to two reporters except adding an extra fluorescent protein; for VLP production and prediction, equations are summarized in Supplementary Table S7. All the modeling and prediction steps were conducted by MATLAB R2016, and parameters were listed in the supplementary tables.

### Statistics and reproducibility

No data were excluded from the analyses. The experiments were not randomized. The investigators were not blinded to allocation during experiments and outcome assessment. No statistical method was used to predetermine sample sizes. But the sample sizes were made sufficient for all conclusions to be statistically significant. The transmission electron microscopy of VLPs was repeated twice independently with similar results. The fluorescent VLP colocalization experiment was repeated at least three times independently with similar results. The experiment of Fig. 1b also was repeated at least three times independently with similar results.

### Reporting summary

Further information on research design is available in the Nature Portfolio Reporting Summary linked to this article.

## Supplementary information

Supplementary Information

Reporting Summary

## Data Availability

All other data generated in this study are available from the corresponding authors upon request. Source data are provided with this paper.

## Source data

source data

## Code availability

All relevant codes are available at http://bdainformatics.org/dataRepository, accession number 01.
