# Supplementary material for: Precise programming of multigene expression stoichiometry in mammalian cells by a modular and programmable transcriptional system
Source: Nat Commun. 2023 Mar 17;14:1500. doi: 10.1038/s41467-023-37244-y (PMC10023750; doi:10.1038/s41467-023-37244-y)
Supplement: Supplementary file 1 — Supplementary Information [file 41467_2023_37244_MOESM1_ESM.pdf]

Supplementary information for

Precise programming of multigene expression stoichiometry in mammalian cells by a modular and programmable transcriptional system.

Chenrui Qin[1,4†], Yanhui Xiang[2†], Jie Liu[2†], Ruilin Zhang[5], Ziming Liu[2], Tingting Li[2], Zhi Sun[3], Xiaoyi Ouyang[1], Yeqing Zong[6], Haoqian M Zhang[6], Qi Ouyang[1], Long Qian[1*] and Chunbo Lou[2,3*]

[*]Correspondence should be addressed to long.qian@pku.edu.cn (L.Q.) and cb.lou@siat.ac.cn (C.L.)

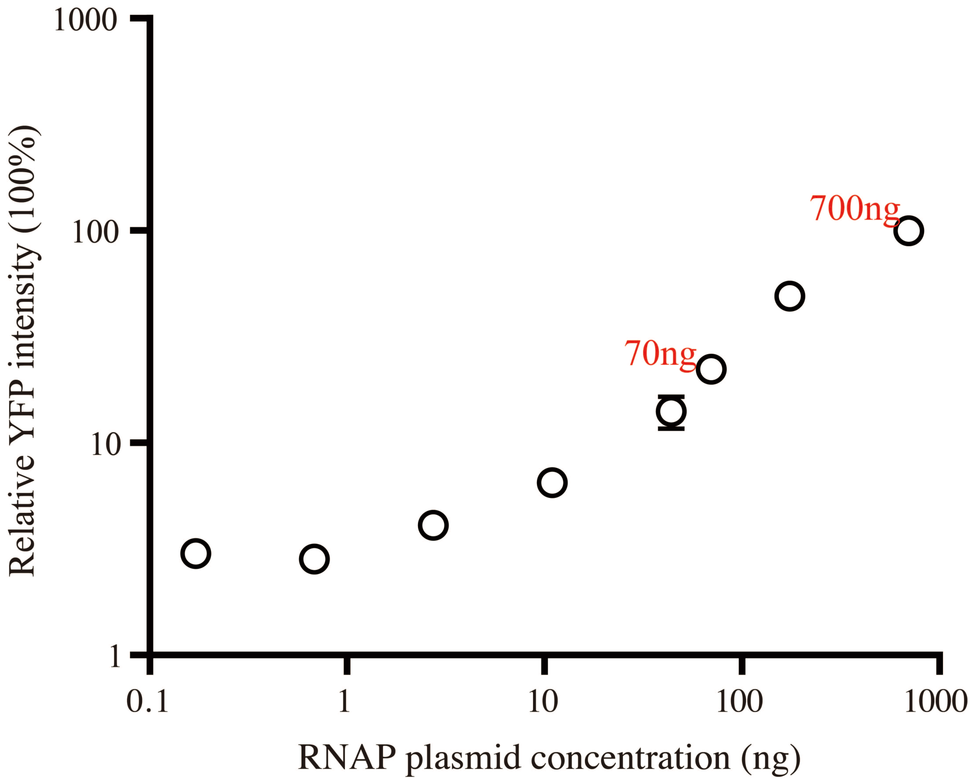

Supplementary Figure S1. The dose response of the orthogonal promoter activity (YFP reporter) to T7RNAP plasmid DNA concentrations. Data represent the mean ±SD (n=4). Source data are provided as a Source Data file.

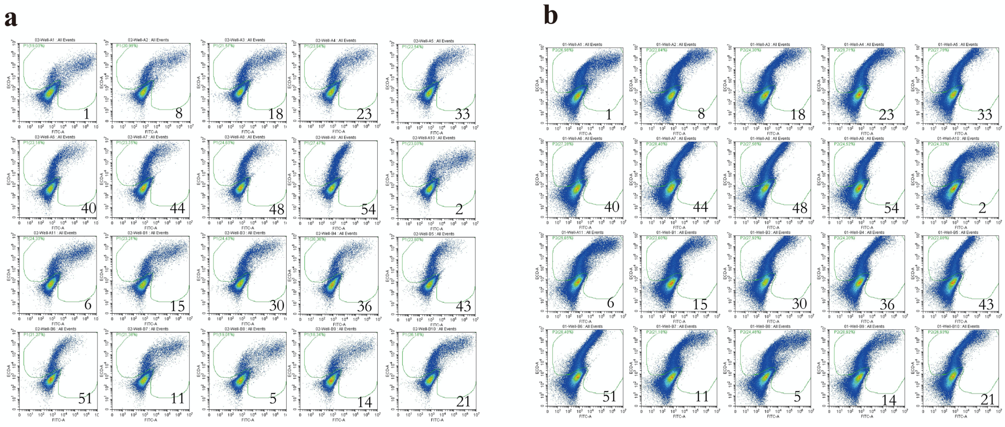

Supplementary Figure S2. Representative FACS plots of Figure1c&d showing the YFP (x-axis, FITC) and mCherry (y-axis, ECD) expression in CHO cells with 0.7 μg (**a**) or 0.07 μg (**b**) T7RNAP plasmid transfection. FACS gating was based on cellular fluorescence relative to non-transfected CHO cells. Yellow fluorescence of positive cells selected (cells in P1 gate) following this pipeline were then analyzed in Figure 1c&d. Constitutively expressed mCherry was also used to determine the transfection efficiency (almost >20%). The index of promotors used were indicated in each plot. Source data are provided as a Source Data file.

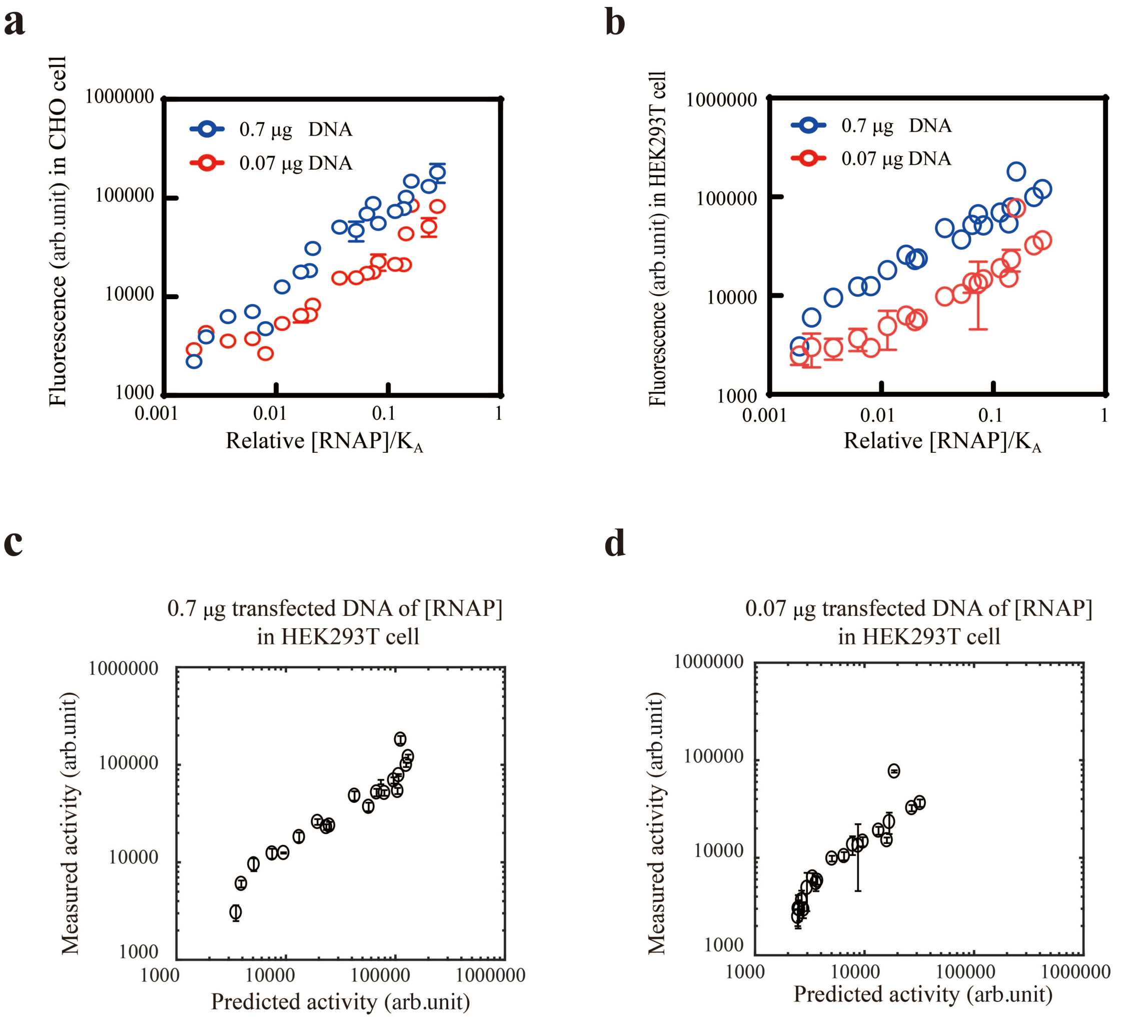

Supplementary Figure S3. The correlations between T7 promoter activities and their pre-determined binding affinities or predicted activities. (**a**) and (**b**) The correlation between T7 promoter activities and their binding affinities under two capping-T7 RNAP expression level in CHO (**a**) and HEK293T (**b**) cells 2 days and 4 days after transfection, respectively. The promoter activity was measured using YFP (Citrine) as the reporter and quantified as the arithmetic mean of flow cytometry fluorescence data. The red and blue circles represent transfection with 0.07 μg and 0.7 μg capping-RNAP plasmid, respectively. The reporter plasmid was transfected at 0.3 μg concentration. (**c**) and (**d**) The correlation between T7 promoter activities in HEK293T cells and the predicated activities from the biochemical model. The capping -RNAP plasmid concentration was 0.7 μg and 0.07 μg in (**c**) and (**d**), respectively. All data represent the mean ± SD (n=3). Source data are provided as a Source Data file.

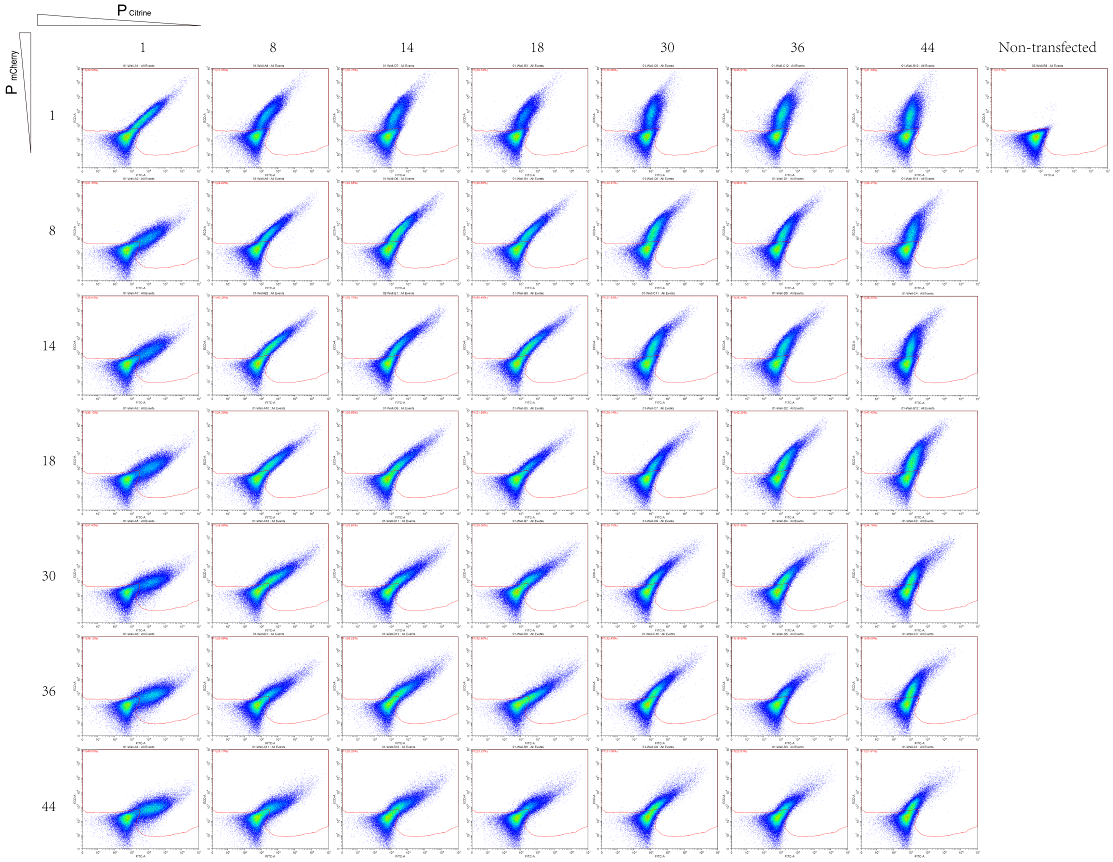

Supplementary Figure S4. Representative FACS plots of the two-reporter system in HEK293T cells. The x- and y-axis showed fluorescence of YFP (Citrine) and mCherry, respectively. These data were used to generate Figure 2b. The indices of promotors used were indicated in each row and each column. Source data (transfection efficacy, cell numbers, raw fluorescence data) are provided as a Source Data file.

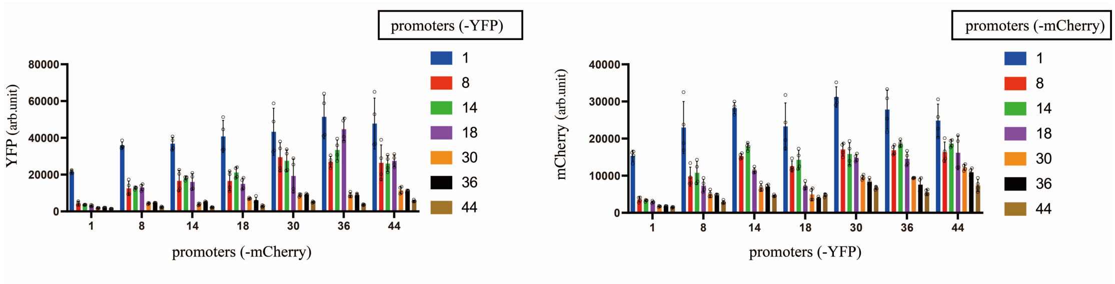

Supplementary Figure S5. Histogram of the heatmap in Figure 2 showing the expression levels of different combinations. Data represent the mean ±SD (n=4). Source data are provided as a Source Data file.

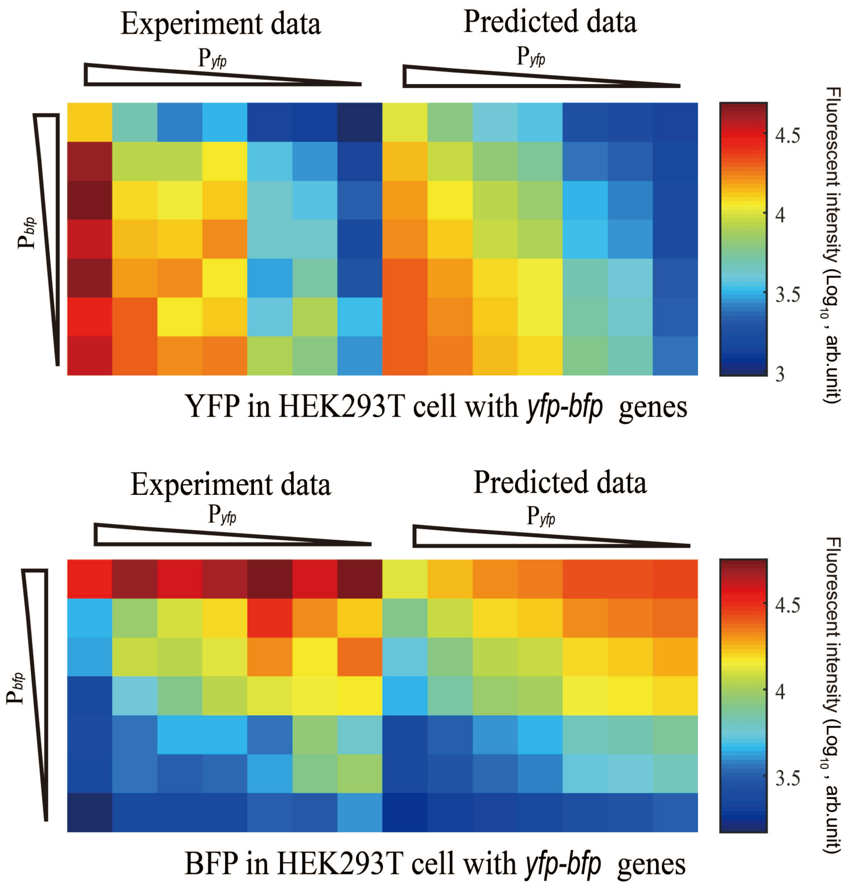

Supplementary Figure S6. Resource competition in the two-reporter system. Experimental data and model prediction of YFP and BFP expression in HEK293T cells transfected with 0.5 µg RNAP plasmid DNA. The orthogonal promoters #1, 8, 14, 18, 30, 36 and 44 were used for the two-reporter systems. Promoter strengths are indicated by the wedged bars.

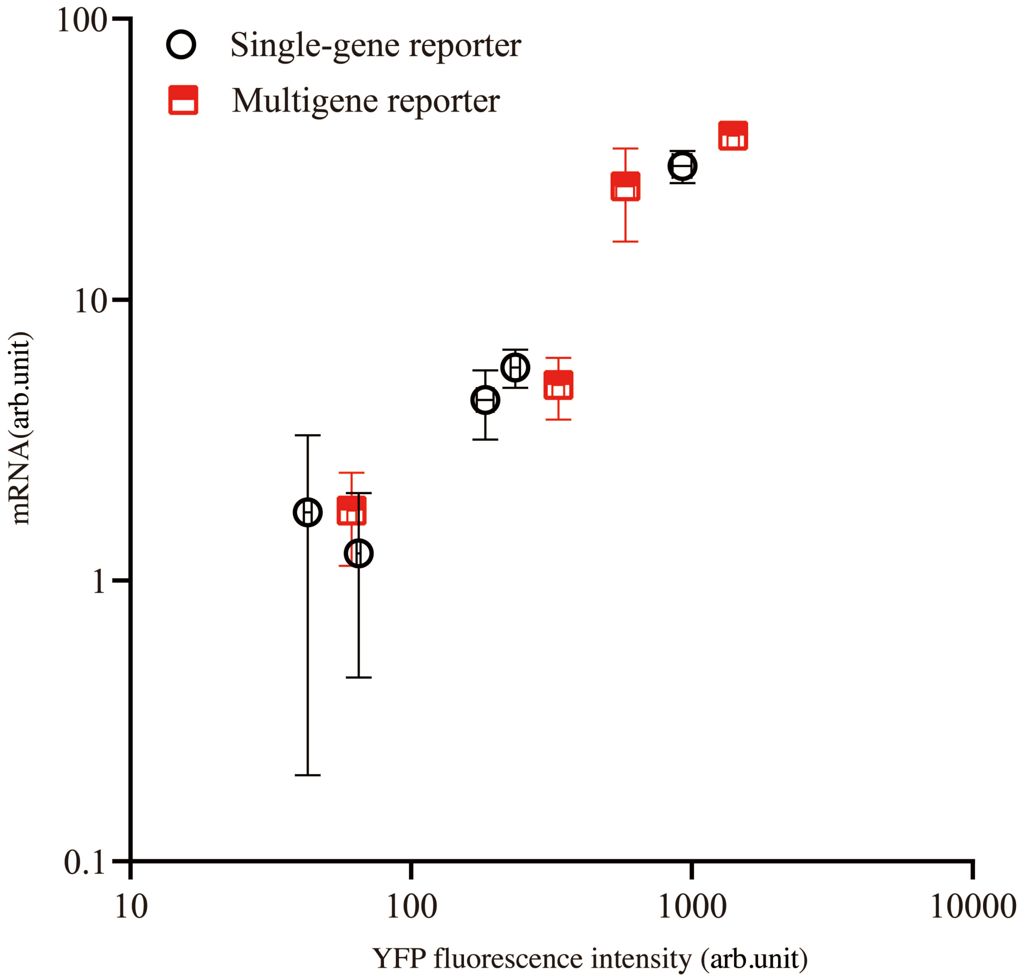

Supplementary Figure S7. Correlation between mRNA transcript levels and reporter expressions in CHO cells transfected with the single- and multiple-gene constructs. Quantitative PCR was used to measure mRNA transcript levels. Data represent the mean ±SD from three biological replicates. R^2 = 0.93.

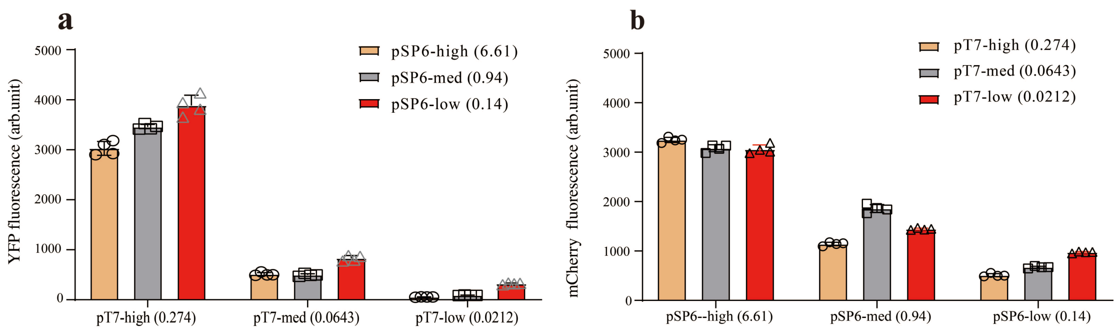

Supplementary Figure S8. Expression of a two-gene system driven by orthogonal RNAPs. Two reporter genes (pT7-YFP and pSP6-mCherry) were transcribed by T7 RNAP and SP6 RNAP. By using orthogonal promoters of high-, medium- and low-activities for both RNAPs, we obtained 3*3=9 constructions. Relative [RNAP]/KA values were indicated in parentheses. The mCherry expression driving by pSP6 promoters with different activities had little influence on YFP expression driving by pT7 promoters(**a**) and vice versa(**b**), indicating that these two types of promoters (pT7 and pSP6) were mostly independent and they did not interfere with each other. Data represent the mean ±SD (n=4). Source data are provided as a Source Data file.

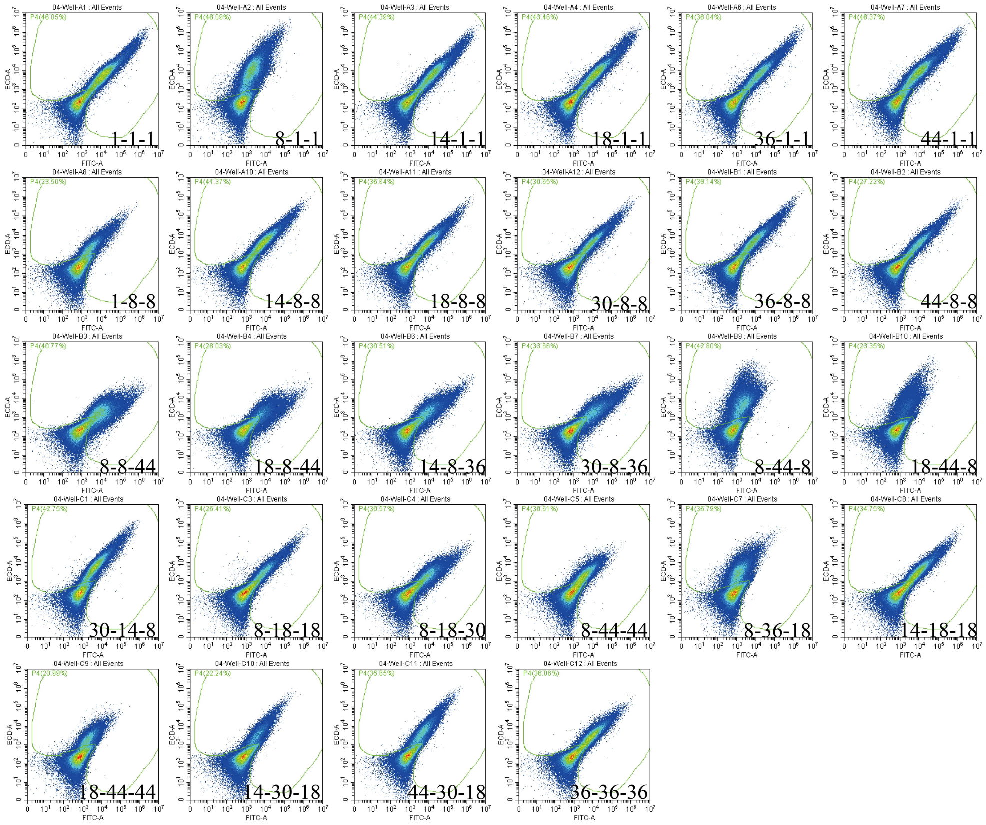

Supplementary Figure S9. Expression of the three-reporter system. Representative FACS plots of Figure 3e showing YFP (x-axis, FITC) and mCherry (y-axis, ECD) expression in HEK293T cells, and the order of the three-reporter system is *bfp-yfp-mCherry*. Source data (raw fluorescence data) are provided as a Source Data file.

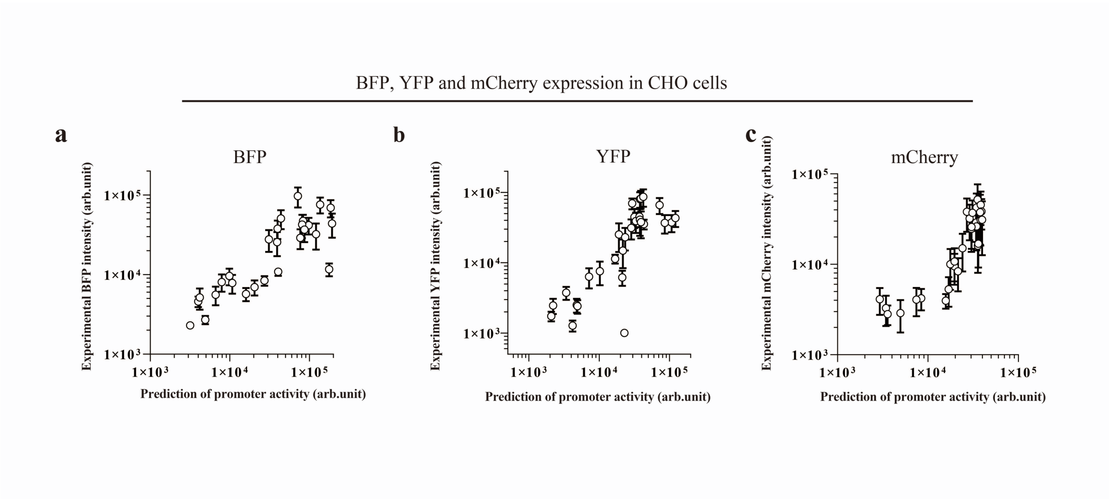

Supplementary Figure S10. The correlation between experimental data and model prediction in the three-reporter system in CHO cells. BFP (**a**), YFP (**b**) and mCherry (**c**) expression levels are extracted from flow cytometry data. Data represent the mean ±SD (n=3).

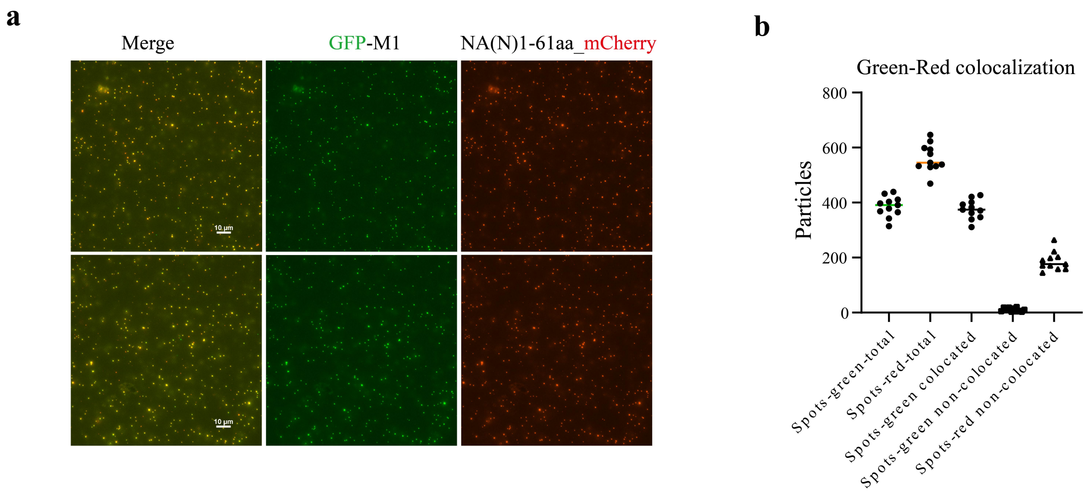

Supplementary Figure S11. Reconstituted VLPs labelled with GFP-M1 and NA-mCherry. **(a)** Representative microscopy images of Fluorescent VLPs. **(b)** The colocalization analysis of GFP-M1 and NA-mCherry in VLPs. Data were collected from a total of 11 microscopy fields and particles were analyzed by the Imaris software (Oxford Instruments).

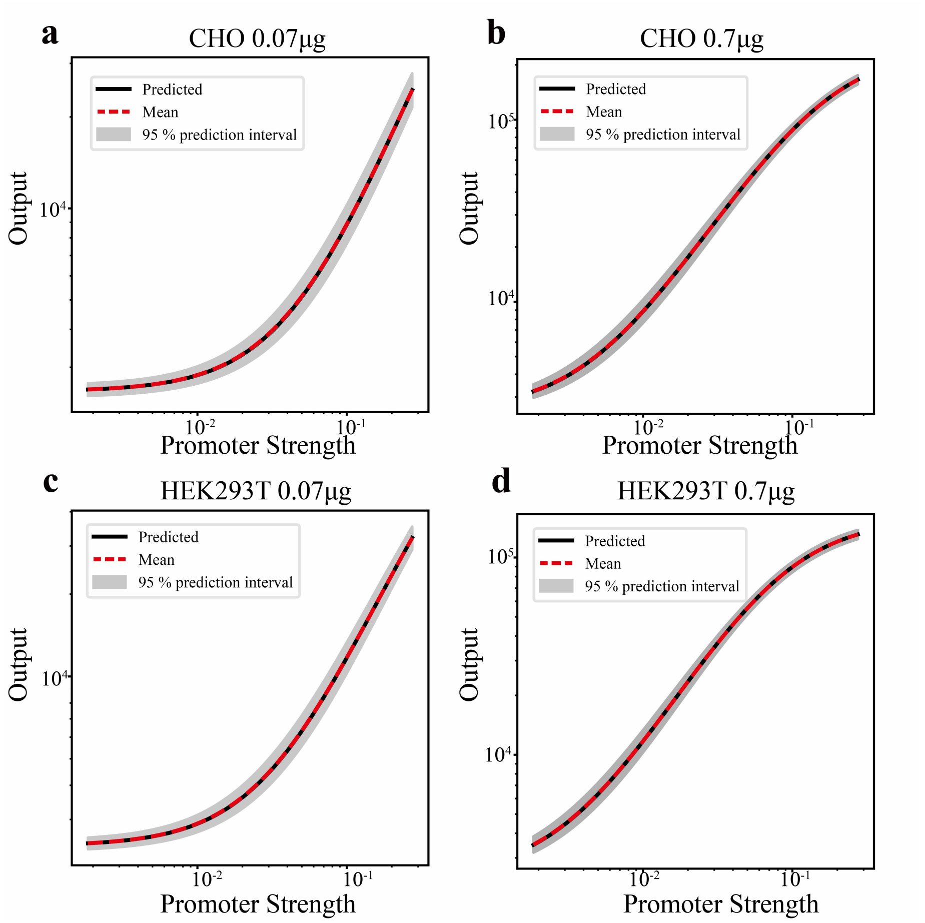

Supplementary Figure S12. Output predictions of four representative one-gene systems with ±5% perturbation in all 4 parameters in the original models. The black curves show the mean predictions, and the shaded area show their 95% prediction intervals (n=40960).

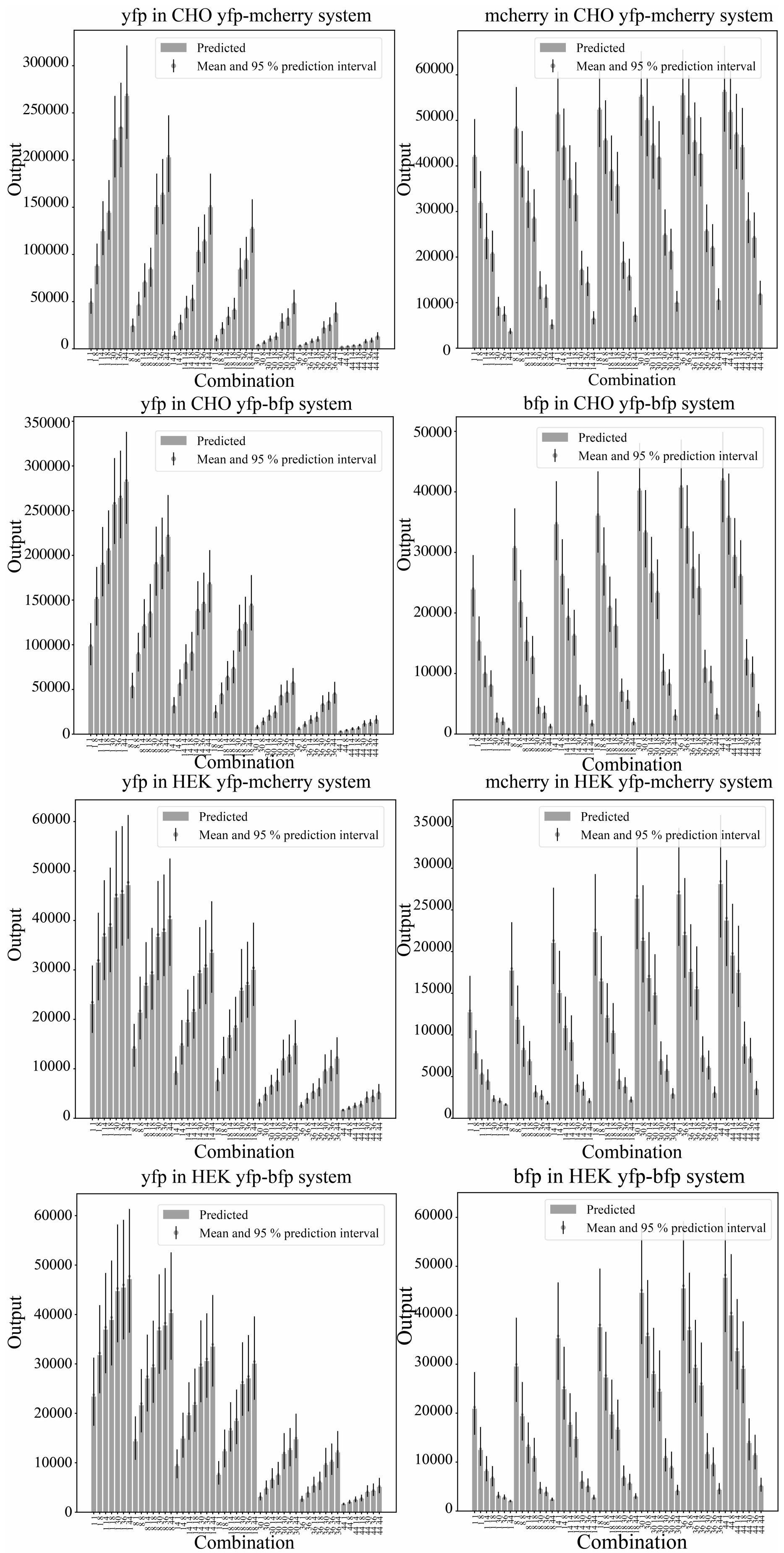
yfp in CHO yfp-mcherry system
Predicted
Mean and 95 % prediction interval
Output
300000
250000
200000
150000
100000
50000
0
Combination
mcherry in CHO yfp-mcherry system
Predicted
Mean and 95 % prediction interval
Output
60000
50000
40000
30000
20000
10000
0
Combination
yfp in CHO yfp-bfp system
Predicted
Mean and 95 % prediction interval
Output
350000
300000
250000
200000
150000
100000
50000
0
Combination
bfp in CHO yfp-bfp system
Predicted
Mean and 95 % prediction interval
Output
50000
40000
30000
20000
10000
0
Combination
yfp in HEK yfp-mcherry system
Predicted
Mean and 95 % prediction interval
Output
60000
50000
40000
30000
20000
10000
0
Combination
mcherry in HEK yfp-mcherry system
Predicted
Mean and 95 % prediction interval
Output
35000
30000
25000
20000
15000
10000
5000
0
Combination
yfp in HEK yfp-bfp system
Predicted
Mean and 95 % prediction interval
Output
60000
50000
40000
30000
20000
10000
0
Combination
bfp in HEK yfp-bfp system
Predicted
Mean and 95 % prediction interval
Output
60000
50000
40000
30000
20000
10000
0
Combination

Supplementary Figure S13. Predicted outputs of four representative two-gene systems (two outputs for each system) with ±5% perturbation in all 7 parameters in the original models. Bars show the mean predictions and error bars show the 95% prediction intervals (n=4608). Numbers below the x-axis represent the rank of promoters used to drive the two genes in each experiment.

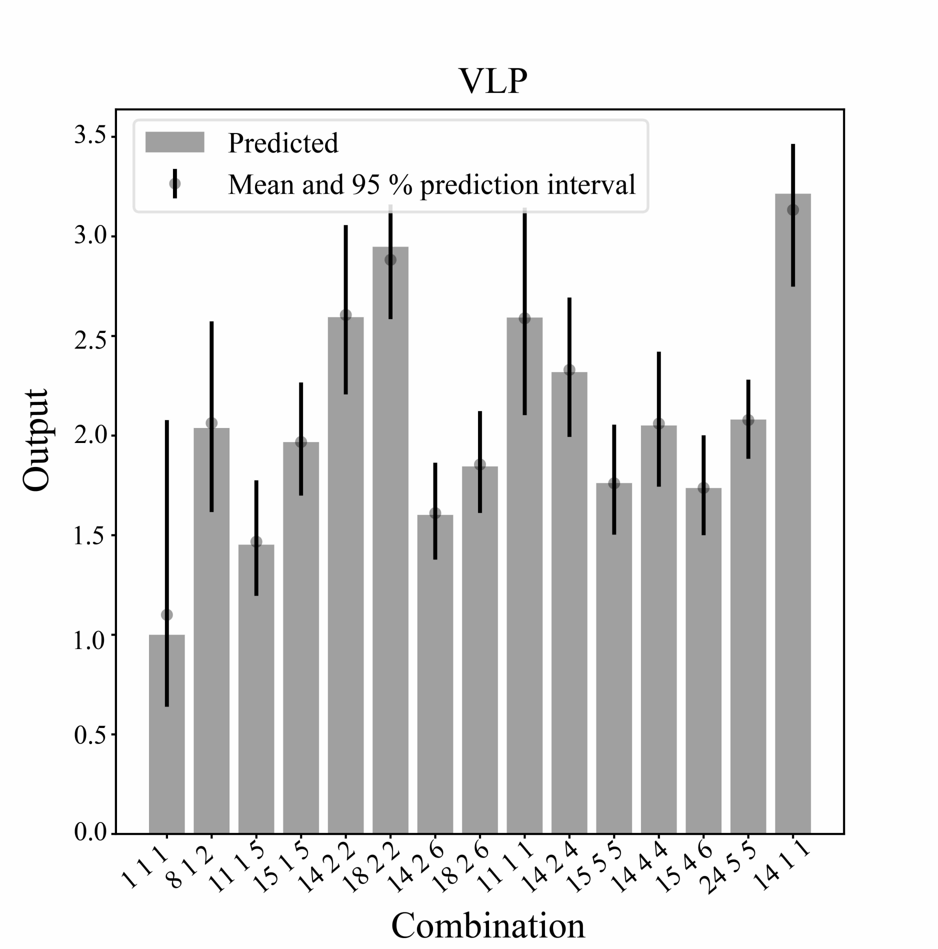

Supplementary Figure S14. Predicted VLP yields of the three-gene system with ±5% perturbation in all 7 parameters in the original models. Bars show the predictions and dots and error bars show the mean and 95% prediction intervals (n=4096). Numbers below the x-axis represent the rank of promoters used to drive the three genes in each experiment.

Supplementary Table S1: Fitted parameters of Equation (1) in the main text for CHO cells and HEK293T cells.

**Parameter**	**Description**	**Value in CHO cells**	**Value in HEK293T cells**
β	the basal expression of the promoters	2500	2400
α	the maximal expression of the promoters	247100	155826
$[RNAP]/K_D$	the relative RNAP concentration to the dissociation constant of the promoters to RNAP	6.1 (for 0.7μg DNA)<br>0.61(for 0.07μg DNA)	12 (for 0.7μg DNA)<br>1.2 (for 0.07μg DNA)

n	the Hill coefficcient of RNAP	1.3	1.3

Supplementary Table S2: Fitted parameters of Equations 2-4 in the main text in CHO cells and HEK293T cells.

**Parameter**	**Description**	**Value in CHO cells**	**Value in HEK293T cells**
[DNA]	Rescaling factor for plasmid concentration in a cell	14.8	11.2
β_{yfp}	the basal expression of the promoter driving *yfp*	1566	1253
$\beta_{mcherry}$	the basal expression of the promoter driving *rfp*	1925	1466
β_{bfp}	the basal expression of the promoter driving *bfp*	2540	1742
α_{yfp}	the maximal expression of the promoter driving *yfp*	701547	969122
$\alpha_{mcherry}$	the maximal expression of the promoter driving *mcherry*	107141	575626
α_{bfp}	the maximal expression of the promoter driving *bfp*	918000	996832
$[RNAP]_{tot}/K_{yfp}$	the relative binding affinities of the promoters driving the subscribed genes to the cognate RNAP	1.29	0.48
$[RNAP]_{tot}/K_{mcherry}$		4.9	0.43
$[RNAP]_{tot}/K_{bfp}$		2.1	0.42
n	the hill coefficient of RNAP	1.3	1.3

Supplementary Table S3. Parameter sensitivity of the models. Analysis was done by SALib package in Python3 with sobol method. The sensitivities of parameter relatives to both outputs in the two-gene systems are analyzed and reported respectively. "ST_conf" is the half length of the 95% confidence interval of the sensitivity. Codes are available.

**One-gene model**		
CHO (0.07μg)		
	ST	ST_conf
α	0.018826	0.002279
β	0.002388	0.00015
[RNAP]/K_D	0.023817	0.00238
n	0.125630	0.012302
CHO (0.7μg)		
	ST	ST_conf
α	0.022766	0.001498
β	0.000036	0.000001
[RNAP]/K_D	0.011415	0.000757
n	0.006353	0.000408
HEK (0.07μg)		
	ST	ST_conf
α	0.019412	0.002162
β	0.001289	0.000087
[RNAP]/K_D	0.022467	0.001922

n	0.062915	0.004762
HEK (0.7μg)		
	ST	ST_conf
α	0.026304	0.002069
β	0.000044	0.000002
[RNAP]/K_D	0.007648	0.000474
n	0.004393	0.000293
**Two-gene model**		
CHO yfp-mcherry system output: yfp		
	ST	ST_conf
[DNA]	0.000686	1.09E-04
β_{yfp}	0.000005	3.39E-07
α_{yfp}	0.021825	2.35E-03
$[RNAP]_{tot}/K_{yfp}$	0.008519	7.47E-04
$[RNAP]_{tot}/K_{mcherry}$	0.003011	3.17E-04
n	0.303472	2.41E-02
CHO yfp-mcherry system output: mcherry		
	ST	ST_conf
[DNA]	0.00142	0.000128
$\beta_{mcherry}$	0.000105	0.000006
$\alpha_{mcherry}$	0.039433	0.002552
$[RNAP]_{tot}/K_{yfp}$	0.001202	0.000115
$[RNAP]_{tot}/K_{mcherry}$	0.005281	0.000343
n	0.427827	0.020714
CHO yfp-bfp system output: yfp		
	ST	ST_conf
[DNA]	0.001119	1.44E-04
β_{yfp}	0.000004	2.33E-07
α_{yfp}	0.024602	2.45E-03
$[RNAP]_{tot}/K_{yfp}$	0.008158	6.56E-04
$[RNAP]_{tot}/K_{bfp}$	0.002003	2.13E-04
n	0.325804	1.92E-02
CHO yfp-bfp system output: bfp		
	ST	ST_conf
[DNA]	0.001195	1.39E-04
β_{bfp}	0.000003	2.05E-07
α_{bfp}	0.029855	2.11E-03
$[RNAP]_{tot}/K_{yfp}$	0.00136	1.19E-04
$[RNAP]_{tot}/K_{bfp}$	0.006728	4.49E-04
n	0.358889	1.88E-02
HEK yfp-mcherry system output: yfp		
	ST	ST_conf
[DNA]	0.000839	0.000114
β_{yfp}	0.00004	0.000002
α_{yfp}	0.024079	0.002726
$[RNAP]_{tot}/K_{yfp}$	0.005539	0.000468
$[RNAP]_{tot}/K_{mcherry}$	0.001495	0.000168
n	0.546854	0.035699

HEK yfp-mcherry system output: mcherry		
	ST	ST_conf
[DNA]	0.000809	0.000122
$\beta_{mcherry}$	0.000172	0.000011
$\alpha_{mcherry}$	0.023318	0.002535
$[RNAP]_{tot}/K_{yfp}$	0.001624	0.000154
$[RNAP]_{tot}/K_{mcherry}$	0.005843	0.00047
n	0.533253	0.038195
HEK yfp-bfp system output: yfp		
	ST	ST_conf
[DNA]	0.00085	0.000118
β_{yfp}	0.00004	0.000003
α_{yfp}	0.024143	0.002453
$[RNAP]_{tot}/K_{yfp}$	0.005526	0.000449
$[RNAP]_{tot}/K_{bfp}$	0.001473	0.000169
n	0.548066	0.037507
HEK yfp-bfp system output: bfp		
	ST	ST_conf
[DNA]	0.000819	0.00012
β_{bfp}	0.000082	0.000005
α_{bfp}	0.023218	0.002309
$[RNAP]_{tot}/K_{yfp}$	0.001633	0.000181
$[RNAP]_{tot}/K_{bfp}$	0.005897	0.000462
n	0.531842	0.032639
**VLP system**		
	ST	ST_conf
[RNAP]	0.300772	0.10799
[DNA]	0.116172	0.044949
$\lg(\alpha_1 / f_1)$	0.006437	0.008557
$\lg(\alpha_2 / f_2)$	0.023761	0.010092
R1	0.013705	0.005633
R2	0.032471	0.020279
R3	0.024959	0.013007

Supplementary Table S4. The sequences of T7 core promoters. Red ones were tested in mammalian cells.

Index/Rank	Sequence	[Activator]/K_A in *E.coli*
1	TAATACGACTCACTATAGGGG	0.274
2	ATTTACGACTCACTATAGGGG	0.23
3	AAATACGACTCACTATAGGGG	0.227
4	GAATACGACTCACTATAGGGG	0.191
5	TAAAACGACTCACTATAGGGG	0.16
6	TTTAACGACTCACTATAGGGG	0.144
7	CAAAACGACTCACTATAGGGG	0.142
8	AATTCCGACTCACTATAGGGG	0.137
9	AAAAACGACTCACTATAGGGG	0.124
10	AGTTACGACTCACTATAGGGG	0.117

11	TATAACGACTCACTATAGGGG	0.115
12	GAAAACGACTCACTATAGGGG	0.104
13	CTAAACGACTCACTATAGGGG	0.0998
14	ACTTACGACTCACTATAGGGG	0.0816
15	TAAACCGACTCACTATAGGGG	0.0731
16	ATAAACGACTCACTATAGGGG	0.0719
17	GTAAACGACTCACTATAGGGG	0.0706
18	AGATACGACTCACTATAGGGG	0.0643
19	AATCACGACTCACTATAGGGG	0.0589
20	TGATACGACTCACTATAGGGG	0.0565
21	AGAAACGACTCACTATAGGGG	0.0518
22	GGATACGACTCACTATAGGGG	0.0382
23	CTACACGACTCACTATAGGGG	0.0367
24	CATCACGACTCACTATAGGGG	0.0332
25	TAATACGACTCAGTCAAGGGG	0.0273
26	ATAAGCGACTCACTATAGGGG	0.0267
27	ATACACGACTCACTATAGGGG	0.0256
28	GCATTCGACTCACTATAGGGG	0.0247
29	ATCTACGACTCACTATAGGGG	0.0244
30	ACCTACGACTCACTATAGGGG	0.0212
31	ACAAACGACTCACTATAGGGG	0.0204
32	TCCTACGACTCACTATAGGGG	0.0203
33	GCCTACGACTCACTATAGGGG	0.0199
34	CACTACGACTCACTATAGGGG	0.0177
35	GGCTACGACTCACTATAGGGG	0.0169
36	TTGAACGACTCACTATAGGGG	0.0166
37	CACAACGACTCACTATAGGGG	0.0161
38	GACAACGACTCACTATAGGGG	0.0146
39	TATCGCGACTCACTATAGGGG	0.0136
40	CTCTGCGACTCACTATAGGGG	0.0113
41	GTGAACGACTCACTATAGGGG	0.00962
42	CGCAACGACTCACTATAGGGG	0.00956
43	CTCATCGACTCACTATAGGGG	0.00804
44	TTTGTCGACTCACTATAGGGG	0.00614
45	AGCCACGACTCACTATAGGGG	0.00611
46	AGGAACGACTCACTATAGGGG	0.00575
47	GTGCACGACTCACTATAGGGG	0.00396
48	TGCAGCGACTCACTATAGGGG	0.00372
49	ATAGACGACTCACTATAGGGG	0.0365
50	AAAGCCGACTCACTATAGGGG	0.0272
51	CCGTTCGACTCACTATAGGGG	0.00237
52	CGCACCGACTCACTATAGGGG	0.0235
53	GGTCGCGACTCACTATAGGGG	0.0231
54	TAATACGACTCACACTCGGGG	0.00185

Supplementary Table S5. The sequences of seven T7 core promoters selected for two-reporter system tests.

Index	Sequence	[Activator]/K_A in *E.coli*
1	TAATACGACTCACTATAGGGG	0.274
8	AATTCCGACTCACTATAGGGG	0.137
14	ACTTACGACTCACTATAGGGG	0.0816
18	AGATACGACTCACTATAGGGG	0.0643
30	ACCTACGACTCACTATAGGGG	0.0212
36	TTGAACGACTCACTATAGGGG	0.0166

44	TTTGTCGACTCACTATAGGGG	0.00614

Supplementary Table S6. The sequences of promoters used in the gene-order experiment.

P_{T7}-YFP-3’UTR-T7 terminator	TAATACGACTCACTATAGGGGCTCGAGAGCCTCGGTACCCGGGTCG<br>AGGTAGGCGTGTACGGTGGGCGCCTATAAAAGCAGAGCTCGTTTAG<br>TGAACCGTCAGATCGCCTGGAGCAATTCCACAACACTTTTGTCTTAT<br>ACCAACTTTCCGTACCACTTCCTACCTCGTAAAAAACTTATCGATGC<br>CACCATGGTGAGCAAGGGCGAGGAGCTGTTCACCGGGGTGGTGCCC<br>ATCCTGGTCGAGCTGGACGGCGACGTAAACGGCCACAAGTTCAGCG<br>TGTCCGGCGAGGGCGAGGGCGATGCCACCTACGGCAAGCTGACCCT<br>GAAGTTCATCTGCACCACCGGCAAGCTGCCCGTGCCCTGGCCCACCC<br>TCGTGACCACCTTCGGCTACGGCCTGATGTGCTTCGCCCGCTACCCC<br>GACCACATGAAGCAGCACGACTTCTTCAAGTCCGCCATGCCCGAAG<br>GCTACGTCCAGGAGCGCACCATCTTCTTCAAGGACGACGGCAACTA<br>CAAGACCCGCGCCGAGGTGAAGTTCGAGGGCGACACCCTGGTGAAC<br>CGCATCGAGCTGAAGGGCATCGACTTCAAGGAGGACGGCAACATCC<br>TGGGGCACAAGCTGGAGTACAACTACAACAGCCACAACGTCTATAT<br>CATGGCCGACAAGCAGAAGAACGGCATCAAGGTGAACTTCAAGATC<br>CGCCACAACATCGAGGACGGCAGCGTGCAGCTCGCCGACCACTACC<br>AGCAGAACACCCCCATCGGCGACGGCCCCGTGCTGCTGCCCGACAA<br>CCACTACCTGAGCTACCAGTCCGCCCTGAGCAAAGACCCCAACGAG<br>AAGCGCGATCACATGGTCCTGCTGGAGTTCGTGACCGCCGCCGGGA<br>TCACTCTCGGCATGGACGAGCTGTACAAGTAAATCGATGCTCGCTTT<br>CTTGCTGTCCAATTTCTATTAAAGGTTCCTTTGTTCCCTAAGTCCAAC<br>TACTAAACTGGGGGATATTATGAAGGGCCTTGAGCATCTGGATTCTG<br>CCTAGATTAATAGATCTCAAAGGCTCTTTTCAGAGCCACCAAAAAA<br>AAAAAAAAAAAAAAAAAAAAAAAAAAAAAAAAAAAGGCCGGCAT<br>GGTCCCAGCCTCCTCGCTGGCGCCGGCTGGGCAACATTCCGAGGGG<br>ACCGTCCCCTCGGTAATGGCGAATGGGACCCATAGCATAACCCCTT<br>GGGGCCTCTAAACGGGTCTTGAGGGGTTTTTTG
p_{T7}-mCherry-3’UTR-T7 terminator	TAATACGACTCACTATAGGGGCTCGAGAGCCTCGGTACCCGGGTCG<br>AGGTAGGCGTGTACGGTGGGCGCCTATAAAAGCAGAGCTCGTTTAG<br>TGAACCGTCAGATCGCCTGGAGCAATTCCACAACACTTTTGTCTTAT<br>ACCAACTTTCCGTACCACTTCCTACCCTCGTAAAAAACTTATCGATG<br>CCACCATGGTGAGCAAGGGCGAGGAGGATAACATGGCCATCATCAA<br>GGAGTTCATGCGCTTCAAGGTGCACATGGAGGGCTCCGTGAACGGC<br>CACGAGTTCGAGATCGAGGGCGAGGGCGAGGGCCGCCCCTACGAG<br>GGCACCCAGACCGCCAAGCTGAAGGTGACCAAGGGTGGCCCCCTGC<br>CCTTCGCCTGGGACATCCTGTCCCCTCAGTTCATGTACGGCTCCAAG<br>GCCTACGTGAAGCACCCCGCCGACATCCCCGACTACTTGAAGCTGTC<br>CTTCCCCGAGGGCTTCAAGTGGGAGCGCGTGATGAACTTCGAGGAC<br>GGCGGCGTGGTGACCGTGACCCAGGACTCCTCCCTGCAGGACGGCG<br>AGTTCATCTACAAGGTGAAGCTGCGCGGCACCAACTTCCCCTCCGAC<br>GGCCCCGTAATGCAGAAGAAGACCATGGGCTGGGAGGCCTCCTCCG<br>AGCGGATGTACCCCGAGGACGGCGCCCTGAAGGGCGAGATCAAGC<br>AGAGGCTGAAGCTGAAGGACGGCGGCCACTACGACGCTGAGGTCA<br>AGACCACCTACAAGGCCAAGAAGCCCGTGCAGCTGCCCGGCGCCTA<br>CAACGTCAACATCAAGTTGGACATCACCTCCCACAACGAGGACTAC<br>ACCATCGTGGAACAGTACGAACGCGCCGAGGGCCGCCACTCCACCG<br>GCGGCATGGACGAGCTGTACAAGTAAATCGATGCTCGCTTTCTTGCT<br>GTCCAATTTCTATTAAAGGTTCCTTTGTTCCCTAAGTCCAACTACTAA<br>ACTGGGGGATATTATGAAGGGCCTTGAGCATCTGGATTCTGCCTAG<br>ATTAATAGATCTCAAAGGCTCTTTTCAGAGCCACCAAAAAAAAAAA<br>AAAAAAAAAAAAAAAAAAAAAAAAAAAAAAAGGCCGGCATGGTCCC<br>AGCCTCCTCGCTGGCGCCGGCTGGGCAACATTCCGAGGGGACCGTC<br>CCCTCGGTAATGGCGAATGGGACCCATAGCATAACCCCTTGGGGCC<br>TCTAAACGGGTCTTGAGGGGTTTTTTG

p_{T7}-BFP-3'UTR-T7 terminator	TAATACGACTCACTATAGGGGCTCGAGAGCCTCGGTACCCGGGTCG<br>AGGTAGGCGTGTACGGTGGGCGCCTATAAAAGCAGAGCTCGTTTAG<br>TGAACCGTCAGATCGCCTGGAGCAATTCCACAACACTTTTGTCTTAT<br>ACCAACTTTCCGTACCACTTCCTACCTCGTAAAAAACTTATCGATGC<br>CACCATGAGCGAGCTGATTAAGGAGAACATGCACATGAAGCTGTAC<br>ATGGAGGGCACCGTGGACAACCATCACTTCAAGTGCACATCCGAGG<br>GCGAAGGCAAGCCCTACGAGGGCACCCAGACCATGAGAATCAAGG<br>TGGTCGAGGGCGGCCCTCTCCCCTTCGCCTTCGACATCCTGGCTACT<br>AGCTTCCTCTACGGCAGCAAGACCTTCATCAACCACACCCAGGGCA<br>TCCCCGACTTCTTCAAGCAGTCCTTCCCTGAGGGCTTCACATGGGAG<br>AGAGTCACCACATACGAAGACGGGGGCGTGCTGACCGCTACCCAGG<br>ACACCAGCCTCCAGGACGGCTGCCTCATCTACAACGTCAAGATCAG<br>AGGGGTGAACTTCACATCCAACGGCCCTGTGATGCAGAAGAAAACA<br>CTCGGCTGGGAGGCCTTCACCGAGACGCTGTACCCCGCTGACGGCG<br>GCCTGGAAGGCAGAAACGACATGGCCCTGAAGCTCGTGGGCGGGA<br>GCCATCTGATCGCAAACATCAAGACCACATATAGATCCAAGAAACC<br>CGCTAAGAACCTCAAGATGCCTGGCGTCTACTATGTGGACTACAGA<br>CTGGAAAGAATCAAGGAGGCCAACAACGAGACCTACGTCGAGCAG<br>CACGAGGTGGCAGTGGCCAGATACTGCGACCTCCCTAGCAAACTGG<br>GGCACAAGCTTAATTAAAGGGCCGCCACTCCACCGGCGGCATGGAC<br>GAGCTGTACAAGTAAATCGATGCTCGCTTTCTTGCTGTCCAATTTCT<br>ATTAAAGGTTCCTTTGTTCCCTAAGTCCAACTACTAAACTGGGGGAT<br>ATTATGAAGGGCCTTGAGCATCTGGATTCTGCCTAGATTAATAGATC<br>TCAAAGGCTCTTTTCAGAGCCACCAAAAAAAAAAAAAAAAAAAA<br>AAAAAAAAAAAAAAAAAAAAGGCCGGCATGGTCCCAGCCTCCTCG<br>CTGGCGCCGGCTGGGCAACATTCCGAGGGGACCGTCCCCTCGGTAA<br>TGGCGAATGGGACCCATAGCATAACCCCTTGGGGCCTCTAAACGGG<br>TCTTGAGGGGTTTTTTG

Supplementary Table S7. The predetermined parameters and the gene-specific parameters in the model predicting VLP yields

$RNAP_{tot}$	DNA_{tot}	$\alpha1/f_1$	$\alpha2/f_2$	$\alpha3/f3$	R_1	R_2	R_3
530.263	553.961	0.0197	0.853	1(normalized)	0.0317	2.775	2.838

$$K_1' = R_1 K_1, \qquad K_2' = R_2 K_2, \qquad K_3' = R_3 K_3$$

$$RNAP_{free} = \frac{RNAP_{tot}}{1 + \left(\frac{DNA_{1\,free}}{K_1'}\right)^{nn} + \left(\frac{DNA_{2\,free}}{K_2'}\right)^{nn} + \left(\frac{DNA_{3\,free}}{K_3'}\right)^{nn}}$$

$$DNA_{1\,free} = \frac{DNA_{1\,tot}}{1 + \left(\frac{RNAP_{free}}{K_1'}\right)^{nn}}$$

$$DNA_{2\,free} = \frac{DNA_{2\,tot}}{1 + \left(\frac{RNAP_{free}}{K_2'}\right)^{nn}}$$

$$DNA_{3\,free} = \frac{DNA_{3\,tot}}{1 + \left(\frac{RNAP_{free}}{K_3'}\right)^{nn}}$$

$$n_{1(HA)} = \frac{1}{f_1} \frac{\alpha_1 DNA_{1\,tot} \left(\frac{RNAP_{free}}{K_1'}\right)^{nn}}{1 + \left(\frac{RNAP_{free}}{K_1'}\right)^{nn}}$$

$$n_{2(M1)} = \frac{1}{f_2} \frac{\alpha_2 DNA_{2\,tot} \left(\frac{RNAP_{free}}{K_2'}\right)^{nn}}{1 + \left(\frac{RNAP_{free}}{K_2'}\right)^{nn}}$$

$$n_{3(NA)} = \frac{1}{f_3} \frac{\alpha_3 DNA_{3\,tot} \left(\frac{RNAP_{free}}{K_3'}\right)^{nn}}{1 + \left(\frac{RNAP_{free}}{K_3'}\right)^{nn}}$$

$$n(VLP) = \min(n_{1(HA)}, n_{2(M1)}, n_{3(NA)})$$

Supplementary Table S8. Predicted VLP yield and observed VLP yield (mean and SD). Values are normalized to the yield of combination (1,1,1) in each set of data. Correlation coefficient between prediction and observation r = 0.70. The promoters are ranked by strength, which are the same as Supplementary Table S3. Fig. 4g and labeled points in Fig. 4f are visualizations of this table.

Combination of promoters			Predicted yield	Observed mean	Observed SD
P1	P2	P3			
0.274 (#1)	0.274 (#1)	0.274 (#1)	100	100	6.865
0.137 (#8)	0.274 (#1)	0.23 (#2)	204.7	221.910	26.348
0.115 (#11)	0.274 (#1)	0.16 (#5)	145.9	153.650	4.322
0.0731 (#15)	0.274 (#1)	0.16 (#5)	195.6	146.327	5.658
0.0816 (#14)	0.23 (#2)	0.23 (#2)	259.2	203.157	22.486
0.0643 (#18)	0.23 (#2)	0.23 (#2)	288.7	202.787	37.781
0.0816 (#14)	0.23 (#2)	0.144 (#6)	160.1	132.653	19.253
0.0643 (#18)	0.23 (#2)	0.144 (#6)	181	157.850	17.778
0.115 (#11)	0.274 (#1)	0.274 (#1)	257.5	185.563	4.322
0.0816 (#14)	0.23 (#2)	0.191 (#4)	231.7	170.133	43.131
0.0731 (#15)	0.16 (#5)	0.16 (#5)	172.1	147.060	27.33
0.0816 (#14)	0.191 (#4)	0.191 (#4)	205	145.197	22.534
0.0731 (#15)	0.191 (#4)	0.144 (#6)	169.7	151.817	15.092
0.0332 (#24)	0.16 (#5)	0.16 (#5)	205.5	229.933	31.412
0.0816 (#14)	0.274 (#1)	0.274 (#1)	311.5	192.958	30.549

Supplementary Table S9 Sequences of other core promoters, proteins and terminators

Name	Sequence
P_{TRE3G}	TAGGCGTGTACGGTGGGCGCCTATAAAAGCAGAGCTCGTTTAGTGAACCGTCAGATCGCCTGGAGCAATTCCACAACACTTTTGTCTTATACCAACTTTCCGTACCACTTCCTACCCTCGTAAA
$P_{T7\ Core}$	TAATACGACTCACTATAGGGG
$P_{SP6\ Core}$	ATTTAGGTGACACTATAGAGGGG
$P_{T3\ Core}$	ATTAACCCTCACTAAAGGGAA
$P_{K1.5\ Core}$	TAATCAGTATTTACTGGACACTATAGAAGGG
$P_{K11\ Core}$	TTCTAATGAATTAGGGCACACTATAGGGAGA
$P_{Phi15\ Core}$	ACCAGATTTAAAAACCCACACAATAGACAGA
$(G4S)_4$ linker	GGAGGAGGAGGTAGTGGAGGAGGAGGTAGTGGAGGAGGAGGTAGTGGAGGAGGAGGTAGT
YFP (citrine)	ATGGTGAGCAAGGGCGAGGAGCTGTTCACCGGGGTGGTGCCCATCCTGGTCGAGCTGGACGGCGACGTAAACGGCCACAAGTTCAGCGTGTCCGGCGAGGGCGAGGGCGATGCCACCTACGGCAAGCTGACCCTGAAGTTCATCTGCACCACCGGCAAGCTGCCCGTGCCCTGGCCCACCCTCGTGACCACCTTCGGCTACGGCCTGATGTGCTTCGCCCGCTACCCCGACCACATGAAGCAGCACGACTTCTTCAAGTCCGCCATGCCCGAAGGCTACG

	TCCAGGAGCGCACCATCTTCTTCAAGGACGACGGCAACTACAAGACC CGCGCCGAGGTGAAGTTCGAGGGCGACACCCTGGTGAACCGCATCG AGCTGAAGGGCATCGACTTCAAGGAGGACGGCAACATCCTGGGGCA CAAGCTGGAGTACAACTACAACAGCCACAACGTCTATATCATGGCCG ACAAGCAGAAGAACGGCATCAAGGTGAACTTCAAGATCCGCCACAA CATCGAGGACGGCAGCGTGCAGCTCGCCGACCACTACCAGCAGAAC ACCCCCATCGGCGACGGCCCCGTGCTGCTGCCCGACAACCACTACCT GAGCTACCAGTCCGCCCTGAGCAAAGACCCCAACGAGAAGCGCGAT CACATGGTCCTGCTGGAGTTCGTGACCGCCGCCGGGATCACTCTCGG CATGGACGAGCTGTACAAGTAA
RFP (mCherry)	ATGGTGAGCAAGGGCGAGGAGGATAACATGGCCATCATCAAGGAGT TCATGCGCTTCAAGGTGCACATGGAGGGCTCCGTGAACGGCCACGAG TTCGAGATCGAGGGCGAGGGCGAGGGCCGCCCCTACGAGGGCACCC AGACCGCCAAGCTGAAGGTGACCAAGGGTGGCCCCCTGCCCTTCGCC TGGGACATCCTGTCCCCTCAGTTCATGTACGGCTCCAAGGCCTACGT GAAGCACCCCGCCGACATCCCCGACTACTTGAAGCTGTCCTTCCCCG AGGGCTTCAAGTGGGAGCGCGTGATGAACTTCGAGGACGGCGGCGT GGTGACCGTGACCCAGGACTCCTCCCTGCAGGACGGCGAGTTCATCT ACAAGGTGAAGCTGCGCGGCACCAACTTCCCCTCCGACGGCCCCGTA ATGCAGAAGAAGACCATGGGCTGGGAGGCCTCCTCCGAGCGGATGT ACCCCGAGGACGGCGCCCTGAAGGGCGAGATCAAGCAGAGGCTGAA GCTGAAGGACGGCGGCCACTACGACGCTGAGGTCAAGACCACCTAC AAGGCCAAGAAGCCCGTGCAGCTGCCCGGCGCCTACAACGTCAACA TCAAGTTGGACATCACCTCCCACAACGAGGACTACACCATCGTGGAA CAGTACGAACGCGCCGAGGGCCGCCACTCCACCGGCGGCATGGACG AGCTGTACAAGTAA
BFP (tagBFP)	ATGAGCGAGCTGATTAAGGAGAACATGCACATGAAGCTGTACATGG AGGGCACCGTGGACAACCATCACTTCAAGTGCACATCCGAGGGCGA AGGCAAGCCCTACGAGGGCACCCAGACCATGAGAATCAAGGTGGTC GAGGGCGGCCCTCTCCCCTTCGCCTTCGACATCCTGGCTACTAGCTTC CTCTACGGCAGCAAGACCTTCATCAACCACACCCAGGGCATCCCCGA CTTCTTCAAGCAGTCCTTCCCTGAGGGCTTCACATGGGAGAGAGTCA CCACATACGAAGACGGGGGCGTGCTGACCGCTACCCAGGACACCAG CCTCCAGGACGGCTGCCTCATCTACAACGTCAAGATCAGAGGGGTGA ACTTCACATCCAACGGCCCTGTGATGCAGAAGAAAACACTCGGCTGG GAGGCCTTCACCGAGACGCTGTACCCCGCTGACGGCGGCCTGGAAGG CAGAAACGACATGGCCCTGAAGCTCGTGGGCGGGAGCCATCTGATC GCAAACATCAAGACCACATATAGATCCAAGAAACCCGCTAAGAACC TCAAGATGCCTGGCGTCTACTATGTGGACTACAGACTGGAAAGAATC AAGGAGGCCAACAACGAGACCTACGTCGAGCAGCACGAGGTGGCAG TGGCCAGATACTGCGACCTCCCTAGCAAACTGGGGCACAAGCTTAAT TAA
Capping enzyme	ATGGCCTCCCTGGACAATCTGGTGGCCCGGTACCAGAGATGCTTTAA TGATCAGTCTCTGAAGAACAGCACCATCGAGCTGGAGATCAGATTCC AGCAGATCAACTTCCTGCTGTTTAAGACCGTGTATGAGGCCCTGGTG GCCCAGGAGATCCCTTCTACAATCTCTCACAGCATCAGATGCATCAA GAAGGTGCACCACGAGAATCACTGTAGGGAGAAGATCCTGCCAAGC GAGAACCTGTACTTTAAGAAGCAGCCTCTGATGTTCTTTAAGTTCTCC GAGCCAGCCTCTCTGGGCTGTAAGGTGAGCCTGGCCATCGAGCAGCC TATCAGGAAGTTTATCCTGGACAGCTCCGTGCTGGTGCGGCTGAAGA ACAGAACCACATTCAGGGTGTCCGAGCTGTGGAAGATCGAGCTGACC ATCGTGAAGCAGCTGATGGGCTCTGAGGTGAGCGCCAAGCTGGCAG CCTTCAAGACCCTGCTGTTTGACACACCCGAGCAGCAGACCACAAAG AATATGATGACACTGATCAACCCTGACGATGAGTACCTGTATGAGAT CGAGATCGAGTACACCGGCAAGCCAGAGTCCCTGACAGCAGCAGAT GTGATCAAGATCAAGAATACCGTGCTGACACTGATCTCTCCCAACCA CCTGATGCTGACCGCCTATCACCAGGCCATCGAGTTTATCGCCTCTCA

	CATCCTGTCTAGCGAGATCCTGCTGGCCAGAATCAAGAGCGGCAAGT<br>GGGGCCTGAAGAGGCTGCTGCCACAGGTGAAGTCCATGACCAAGGC<br>CGATTACATGAAGTTCTATCCCCCTGTGGGCTACTATGTGACCGACA<br>AGGCCGATGGCATCCGCGGCATCGCCGTGATCCAGGACACACAGATC<br>TACGTGGTGGCCGATCAGCTGTATAGCCTGGGCACCACAGGCATCGA<br>GCCACTGAAGCCCACCATCCTGGACGGCGAGTTTATGCCCGAGAAGA<br>AGGAGTTCTACGGCTTTGATGTGATCATGTATGAGGGCAATCTGCTG<br>ACCCAGCAGGGCTTCGAGACACGGATCGAGTCCCTGTCTAAGGGCAT<br>CAAGGTGCTGCAGGCCTTTAACATCAAGGCCGAGATGAAGCCCTTCA<br>TCTCCCTGACCTCTGCCGACCCTAACGTGCTGCTGAAGAATTTCGAG<br>AGCATCTTCAAGAAGAAGACCCGCCCTTACTCCATCGATGGCATCAT<br>CCTGGTGGAGCCAGGCAATTCCTATCTGAACACCAATACCTTCAAGT<br>GGAAGCCAACCTGGGACAATACACTGGATTTCCTGGTGCGGAAGTGC<br>CCCGAGTCTCTGAACGTGCCTGAGTACGCCCCAAAGAAGGGCTTTTC<br>TCTGCACCTGCTGTTCGTGGGCATCAGCGGCGAGCTGTTTAAGAAGC<br>TGGCCCTGAACTGGTGTCCAGGCTACACCAAGCTGTTCCCCGTGACA<br>CAGAGAAACCAGAATTATTTCCCCGTGCAGTTTCAGCCCTCTGACTTC<br>CCTCTGGCCTTTCTGTACTATCACCCTGACACCTCCTCTTTCAGCAAT<br>ATCGATGGCAAGGTGCTGGAGATGCGCTGCCTGAAGCGGGAGATCA<br>ACTATGTGAGGTGGGAGATCGTGAAGATCAGGGAGGACCGCCAGCA<br>GGATCTGAAGACAGGCGGCTACTTCGGCAATGACTTTAAGACCGCCG<br>AGCTGACATGGCTGAACTATATGGACCCCTTCAGCTTCGAGGAGCTG<br>GCCAAGGGCCCCTCTGGCATGTACTTTGCCGGCGCCAAGACCGGCAT<br>CTATAGGGCCCAGACAGCCCTGATCTCTTTCATCAAGCAGGAGATCA<br>TCCAGAAGATCAGCCACCAGTCCTGGGTCATCGACCTGGGAATCGGC<br>AAGGGACAGGACCTGGGCAGATACCTGGATGCAGGCGTGAGGCACC<br>TGGTGGGAATCGACAAGGATCAGACCGCCCTGGCAGAGCTGGTGTA<br>CAGGAAGTTCAGCCACGCCACCACACGGCAGCACAAGCACGCCACA<br>AATATCTATGTGCTGCACCAGGATCTGGCCGAGCCTGCCAAGGAGAT<br>CAGCGAGAAGGTGCACCAGATCTACGGCTTTCCAAAGGAGGGCGCC<br>AGCTCCATCGTGTCCAACCTGTTCATCCACTATCTGATGAAGAATACC<br>CAGCAGGTGGAGAACCTGGCCGTGCTGTGCCACAAGCTGCTGCAGCC<br>AGGAGGAATGGTGTGGTTCACCACAATGCTGGGCGAGCAGGTGCTG<br>GAGCTGCTGCACGAGAACCGCATCGAGCTGAATGAAGTGTGGGAGG<br>CCCGGGAGAACGAGGTGGTGAAGTTTGCCATCAAGCGCCTGTTCAAG<br>GAGGACATCCTGCAGGAGACCGGACAGGAGATCGGCGTGCTGCTGC<br>CCTTCTCCAACGGCGACTTCTACAATGAGTATCTGGTGAACACAGCC<br>TTCCTGATCAAGATCTTTAAGCACCACGGCTTCAGCCTGGTGCAGAA<br>GCAGTCCTTCAAGGACTGGATTCCCGAGTTCCAGAACTTCAGCAAGT<br>CCCTGTACAAGATCCTGACCGAGGCCGATAAGACCTGGACAAGCCTG<br>TTCGGCTTTATCTGTCTGCGGAAGAACTGA
T7 RNAP	ATGAACACGATTAACATCGCTAAGAACGACTTCTCTGACATCGAACT<br>GGCTGCTATCCCGTTCAACACTCTGGCTGACCATTACGGTGAGCGTTT<br>AGCTCGCGAACAGTTGGCCCTTGAGCATGAGTCTTACGAGATGGGTG<br>AAGCACGCTTCCGCAAGATGTTTGAGCGTCAACTTAAAGCTGGTGAG<br>GTTGCGGATAACGCTGCCGCCAAGCCTCTCATCACTACCCTACTCCCT<br>AAGATGATTGCACGCATCAACGACTGGTTTGAGGAAGTGAAAGCTA<br>AGCGCGGCAAGCGCCCGACAGCCTTCCAGTTCCTGCAAGAAATCAAG<br>CCGGAAGCCGTAGCGTACATCACCATTAAGACCACTCTGGCTTGCCT<br>AACCAGTGCTGACAATACAACCGTTCAGGCTGTAGCAAGCGCAATCG<br>GTCGGGCCATTGAGGACGAGGCTCGCTTCGGTCGTATCCGTGACCTT<br>GAAGCTAAGCACTTCAAGAAAAACGTTGAGGAACAACTCAACAAGC<br>GCGTAGGGCACGTCTACAAGAAAGCATTTATGCAAGTTGTCGAGGCT<br>GACATGCTCTCTAAGGGTCTACTCGGTGGCGAGGCGTGGTCTTCGTG<br>GCATAAGGAAGACTCTATTCATGTAGGAGTACGCTGCATCGAGATGC<br>TCATTGAGTCAACCGGAATGGTTAGCTTACACCGCCAAAATGCTGGC<br>GTAGTAGGTCAAGACTCTGAGACTATCGAACTCGCACCTGAATACGC

	TGAGGCTATCGCAACCCGTGCAGGTGCGCTGGCTGGCATCTCTCCGA<br>TGTTCCAACCTTGCGTAGTTCCTCCTAAGCCGTGGACTGGCATTACTG<br>GTGGTGGCTATTGGGCTAACGGTCGTCGTCCTCTGGCGCTGGTGCGT<br>ACTCACAGTAAGAAAGCACTGATGCGCTACGAAGACGTTTACATGCC<br>TGAGGTGTACAAAGCGATTAACATTGCGCAAAACACCGCATGGAAA<br>ATCAACAAGAAAGTCCTAGCGGTCGCCAACGTAATCACCAAGTGGA<br>AGCATTGTCCGGTCGAGGACATCCCTGCGATTGAGCGTGAAGAACTC<br>CCGATGAAACCGGAAGACATCGACATGAATCCTGAGGCTCTCACCGC<br>GTGGAAACGTGCTGCCGCTGCTGTGTACCGCAAGGACAAGGCTCGCA<br>AGTCTCGCCGTATCAGCCTTGAGTTCATGCTTGAGCAAGCCAATAAG<br>TTTGCTAACCATAAGGCCATCTGGTTCCCTTACAACATGGACTGGCG<br>CGGTCGTGTTTACGCTGTGTCAATGTTCAACCCGCAAGGTAACGATA<br>TGACCAAAGGACTGCTTACGCTGGCGAAAGGTAAACCAATCGGTAA<br>GGAAGGTTACTACTGGCTGAAAATCCACGGTGCAAACTGTGCGGGTG<br>TCGATAAGGTTCCGTTCCCTGAGCGCATCAAGTTCATTGAGGAAAAC<br>CACGAGAACATCATGGCTTGCGCTAAGTCTCCACTGGAGAACACTTG<br>GTGGGCTGAGCAAGATTCTCCGTTCTGCTTCCTTGCGTTCTGCTTTGA<br>GTACGCTGGGGTACAGCACCACGGCCTGAGCTATAACTGCTCCCTTC<br>CGCTGGCGTTTGACGGGTCTTGCTCTGGCATCCAGCACTTCTCCGCGA<br>TGCTCCGAGATGAGGTAGGTGGTCGCGCGGTTAACTTGCTTCCTAGT<br>GAAACCGTTCAGGACATCTACGGGATTGTTGCTAAGAAAGTCAACGA<br>GATTCTACAAGCAGACGCAATCAATGGGACCGATAACGAAGTAGTT<br>ACCGTGACCGATGAGAACACTGGTGAAATCTCTGAGAAAGTCAAGCT<br>GGGCACTAAGGCACTGGCTGGTCAATGGCTGGCTTACGGTGTTACTC<br>GCAGTGTGACTAAGCGTTCAGTCATGACGCTGGCTTACGGGTCCAAA<br>GAGTTCGGCTTCCGTCAACAAGTGCTGGAAGATACCATTCAGCCAGC<br>TATTGATTCCGGCAAGGGTCTGATGTTCACTCAGCCGAATCAGGCTG<br>CTGGATACATGGCTAAGCTGATTTGGGAATCTGTGAGCGTGACGGTG<br>GTAGCTGCGGTTGAAGCAATGAACTGGCTTAAGTCTGCTGCTAAGCT<br>GCTGGCTGCTGAGGTCAAAGATAAGAAGACTGGAGAGATTCTTCGCA<br>AGCGTTGCGCTGTGCATTGGGTAACTCCTGATGGTTTCCCTGTGTGGC<br>AGGAATACAAGAAGCCTATTCAGACGCGCTTGAACCTGATGTTCCTC<br>GGTCAGTTCCGCTTACAGCCTACCATTAACACCAACAAAGATAGCGA<br>GATTGATGCACACAAACAGGAGTCTGGTATCGCTCCTAACTTTGTAC<br>ACAGCCAAGACGGTAGCCACCTTCGTAAGACTGTAGTGTGGGCACAC<br>GAGAAGTACGGAATCGAATCTTTTGCACTGATTCACGACTCCTTCGG<br>TACCATTCCGGCTGACGCTGCGAACCTGTTCAAAGCAGTGCGCGAAA<br>CTATGGTTGACACATATGAGTCTTGTGATGTACTGGCTGATTTCTACG<br>ACCAGTTCGCTGACCAGTTGCACGAGTCTCAATTGGACAAAATGCCA<br>GCACTTCCGGCTAAAGGTAACTTGAACCTCCGTGACATCTTAGAGTC<br>GGACTTCGCGTTCGCGTAA
SP6 RNAP	ATGCAGGACCTGCACGCTATTCAGCTGCAACTGGAGGAGGAGATGTT<br>CAACGGGGGGATTAGAAGATTCGAGGCCGATCAGCAGAGGCAGATC<br>GCCGCCGGCTCTGAGAGCGACACCGCATGGAACCGGAGACTGCTGTC<br>CGAGCTGATCGCCCCTATGGCCGAGGGCATCCAGGCCTACAAGGAG<br>GAGTATGAGGGCAAGAAGGGAAGGGCCCCACGCGCCCTGGCCTTCC<br>TCCAGTGCGTGGAGAACGAGGTGGCCGCCTACATCACAATGAAGGT<br>GGTCATGGACATGCTGAATACCGATGCCACCCTCCAGGCAATCGCAA<br>TGTCCGTGGCAGAGCGGATCGAGGATCAGGTGAGATTCTCTAAGCTG<br>GAGGGCCACGCCGCCAAGTACTTTGAGAAGGTGAAGAAGAGCCTGA<br>AGGCCTCCAGGACCAAGTCTTATAGGCACGCACACAACGTGGCAGTG<br>GTGGCAGAGAAGTCTGTGGCCGAGAAGGACGCCGATTTCGACAGAT<br>GGGAGGCCTGGCCAAAGGAGACACAGCTCCAGATCGGCACCACACT<br>GCTGGAGATCCTGGAGGGCAGCGTGTTCTACAATGGCGAGCCCGTGT<br>TTATGCGGGCCATGAGAACCTATGGCGGCAAGACAATCTACTACCTC<br>CAGACCAGCGAGTCTGTGGGCCAGTGGATCTCTGCCTTTAAGGAGCA<br>CGTGGCACAGCTGAGCCCTGCATACGCACCATGCGTGATCCCACCTA

	GGCCCTGGAGGACCCCTTTCAACGGAGGATTTCACACAGAGAAGGTG<br>GCCAGCCGGATCAGACTGGTGAAGGGCAATCGGGAGCACGTGAGAA<br>AGCTGACCCAGAAGCAGATGCCCAAGGTGTACAAGGCCATCAACGC<br>CCTCCAGAATACACAGTGGCAGATCAACAAGGACGTGCTGGCCGTG<br>ATCGAGGAAGTGATCCGCCTGGATCTGGGCTATGGCGTGCCCAGCTT<br>CAAGCCCCTGATCGACAAGGAGAACAAGCCCGCCAATCCAGTGCCC<br>GTGGAGTTTCAGCACCTGCGGGGCAGAGAGCTGAAGGAGATGCTGT<br>CCCCTGAGCAGTGGCAGCAGTTCATCAATTGGAAGGGAGAGTGCGC<br>AAGGCTGTATACCGCCGAGACAAAGAGAGGCAGCAAGTCCGCCGCC<br>GTGGTGAGGATGGTGGGACAGGCCCGCAAGTACAGCGCCTTCGAGT<br>CCATCTACTTCGTGTACGCCATGGACTCTAGGAGCCGCGTGTATGTG<br>CAGAGCAGCACCCTGTCCCCACAGTCTAACGATCTGGGCAAGGCCCT<br>GCTGCGGTTCACAGAGGGCAGACCCGTGAATGGCGTGGAGGCCCTG<br>AAGTGGTTTTGTATCAACGGCGCCAATCTGTGGGGCTGGGACAAGAA<br>GACCTTCGATGTGAGGGTGAGCAACGTGCTGGACGAGGAGTTTCAGG<br>ATATGTGCAGGGACATCGCCGCCGATCCCCTGACCTTCACACAGTGG<br>GCCAAGGCCGATGCCCCTTACGAGTTCCTGGCCTGGTGTTTTGAGTA<br>CGCCCAGTATCTGGATCTGGTGGACGAGGGCAGGGCCGACGAGTTTC<br>GCACCCACCTGCCTGTGCACCAGGATGGCTCCTGCTCTGGCATCCAG<br>CACTATAGCGCCATGCTGAGGGACGAAGTGGGAGCAAAGGCAGTGA<br>ACCTGAAGCCTTCCGACGCCCCACAGGATATCTACGGCGCCGTGGCC<br>CAGGTGGTCATCAAGAAGAACGCCCTGTATATGGACGCCGACGATGC<br>CACCACATTCACCAGCGGCTCCGTGACCCTGTCTGGAACAGAGCTGA<br>GGGCAATGGCATCTGCCTGGGATAGCATCGGCATCACCAGAAGCCTG<br>ACAAAGAAGCCCGTGATGACACTGCCTTACGGCAGCACCAGGCTGA<br>CATGTCGCGAGTCCGTGATCGACTATATCGTGGATCTGGAGGAGAAG<br>GAGGCACAGAAGGCAGTGGCAGAGGGAAGGACCGCCAACAAGGTG<br>CACCCTTTTGAGGACGATAGGCAGGACTACCTGACCCCAGGAGCAGC<br>ATACAATTATATGACAGCCCTGATCTGGCCATCTATCAGCGAGGTGG<br>TGAAGGCCCCCATCGTGGCCATGAAGATGATCCGGCAGCTGGCCAGA<br>TTCGCCGCCAAGAGAAACGAGGGCCTGATGTATACCCTGCCTACAGG<br>CTTTATCCTGGAGCAGAAGATCATGGCCACCGAGATGCTGCGGGTGA<br>GAACATGCCTGATGGGCGATATCAAGATGTCCCTCCAGGTGGAGACA<br>GATATCGTGGACGAGGCAGCAATGATGGGAGCTGCCGCCCCAAATTT<br>CGTGCACGGCCACGACGCCTCTCACCTGATCCTGACCGTGTGCGAGC<br>TGGTGGATAAGGGCGTGACATCCATCGCCGTGATCCACGACTCTTTT<br>GGCACCCACGCCGATAACACCCTGACACTGAGGGTGGCCCTGAAGG<br>GACAGATGGTGGCCATGTACATCGACGGCAATGCCCTCCAGAAGCTG<br>CTGGAGGAGCACGAGGAGAGATGGATGGTGGATACAGGCATCGAGG<br>TGCCAGAGCAGGGCGAGTTTGACCTGAATGAGATTATGGACAGTGA<br>ATACGTCTTTGCCTAA
T3 RNAP	ATGAATATCATCGAGAACATTGAGAAAAACGATTTCAGCGAGATCG<br>AGCTGGCCGCCATCCCCTTCAACACCCTGGCTGACCACTACGGCTCC<br>GCCCTGGCCAAGGAGCAGCTGGCTCTGGAGCACGAGTCCTACGAGTT<br>GGGCGAGAGAAGATTCCTGAAGATGCTGGAAAGACAGGCCAAAGCC<br>GGCGAGATCGCCGACAACGCCGCCGCTAAGCCACTGCTGGCCACCCT<br>GCTGCCCAAGCTGACAACAAGAATCGTGGAATGGCTGGAGGAATAC<br>GCCTCTAAGAAGGGCCGGAAGCCTTCCGCTTATGCCCCTCTGCAACT<br>GTTGAAGCCAGAAGCCTCTGCCTTTATCACACTGAAAGTGATCCTTG<br>CCAGTCTGACCAGCACAAATATGACCACCATCCAGGCCGCCGCCGGC<br>ATGCTGGGCAAGGCCATCGAAGATGAGGCCAGATTTGGCAGAATCA<br>GAGATCTGGAAGCCAAGCACTTCAAGAAGCACGTAGAGGAACAGCT<br>GAACAAGCGGCACGGCCAGGTGTACAAGAAAGCCTTCATGCAGGTG<br>GTGGAAGCCGATATGATAGGCAGAGGCCTGCTGGGAGGCGAGGCCT<br>GGAGCAGCTGGGATAAGGAAACAACCATGCATGTGGGCATCCGGCT<br>CATCGAGATGCTGATCGAGTCTACCGGCCTCGTGGAGCTGCAAAGAC<br>ACAACGCCGGCAACGCCGGCTCTGATCACGAGGCTCTGCAACTGGCC

	CAGGAGTACGTGGACGTTCTGGCTAAAAGAGCCGGAGCCCTGGCCG<br>GAATATCCCCTATGTTCCAGCCGTGCGTGGTTCCTCCTAAGCCTTGGG<br>TCGCCATTACAGGCGGCGGCTACTGGGCTAACGGCAGACGACCTCTG<br>GCTCTGGTTAGAACCCACAGCAAGAAGGGACTGATGCGCTACGAAG<br>ACGTGTACATGCCCGAGGTGTACAAGGCTGTGAATCTCGCCCAGAAC<br>ACCGCCTGGAAGATCAACAAGAAAGTGCTGGCTGTGGTCAATGAGA<br>TCGTGAACTGGAAGAACTGTCCTGTTGCCGATATCCCTAGCCTGGAA<br>CGACAGGAGCTGCCCCCTAAACCCGACGACATCGACACCAACGAGG<br>CCGCCCTTAAGGAATGGAAGAAAGCCGCCGCCGGAATCTACCGGCT<br>GGACAAGGCTAGAGTGTCTAGGAGAATCAGCCTGGAGTTCATGCTGG<br>AGCAGGCCAACAAGTTCGCCTCCAAGAAGGCCATTTGGTTTCCCTAC<br>AACATGGACTGGCGGGGCCGGGTGTATGCCGTGCCCATGTTTAACCC<br>CCAGGGCAACGATATGACTAAGGGCCTGCTGACCCTGGCCAAAGGG<br>AAGCCAATCGGAGAAGAGGGCTTCTACTGGCTGAAGATCCACGGCG<br>CCAATTGCGCCGGAGTGGACAAAGTGCCTTTTCCTGAGAGAATCGCC<br>TTCATCGAGAAGCACGTGGACGACATCCTGGCTTGTGCCAAGGACCC<br>CATTAACAACACCTGGTGGGCCGAACAGGATAGCCCTTTCTGCTTCC<br>TGGCCTTTTGCTTCGAATACGCCGGCGTGACTCACCACGGACTGAGC<br>TACAACTGTAGCCTGCCCCTGGCATTCGACGGAAGCTGTAGCGGCAT<br>CCAGCACTTTAGCGCCATGCTGCGGGACGAGGTGGGCGGCAGAGCC<br>GTGAACCTGCTGCCTTCTGAAACCGTGCAGGACATCTACGGCATCGT<br>GGCCCAAAAGGTGAATGAGATCCTGAAACAGGACGCCATCAACGGC<br>ACCCCTAATGAAATGATCACCGTGACAGACAAGGACACAGGCGAAA<br>TCAGCGAGAAGCTCAAGCTGGGCACCAGCACATTGGCACAACAGTG<br>GCTGGCCTACGGCGTGACCAGAAGCGTGACCAAGCGGTCCGTGATG<br>ACCCTGGCTTACGGCAGCAAGGAGTTCGGCTTCAGACAGCAGGTCCT<br>GGATGACACCATCCAACCTGCTATTGACTCTGGCAAAGGCCTGATGT<br>TCACCCAGCCTAACCAGGCCGCTGGATATATGGCCAAGCTGATCTGG<br>GATGCCGTCAGCGTGACCGTGGTGGCTGCTGTGGAGGCCATGAACTG<br>GCTGAAAAGCGCCGCCAAGCTGCTGGCCGCTGAAGTGAAGGACAAG<br>AAGACCAAGGAAATCCTGAGACACAGATGCGCCGTGCACTGGACCA<br>CCCCTGATGGATTCCCAGTGTGGCAGGAGTATAGAAAGCCTCTGCAA<br>AAGCGGCTGGACATGATTTTCTTAGGACAGTTCAGACTGCAACCTAC<br>CATCAACACACTGAAAGACTCTGGCATCGACGCTCATAAGCAGGAG<br>AGCGGCATCGCCCCTAACTTCGTGCACAGCCAGGACGGCAGCCACCT<br>GAGAATGACCGTGGTGTACGCCCACGAAAAATACGGCATCGAGAGC<br>TTCGCTCTGATCCATGATAGCTTTGGCACAATCCCCGCCGACGCCGG<br>CAAACTGTTCAAGGCCGTGCGGGAAACCATGGTGATCACATACGAA<br>AACAATGACGTGCTGGCCGACTTCTACAGCCAGTTCGCCGACCAGCT<br>GCACGAGACACAGCTGGATAAGATGCCACCTCTGCCTAAGAAGGGG<br>AACCTAAATCTGCAAGACATCCTGAAGAGTGACTTCGCCTTCGCCTG<br>A
K1.5 RNAP	ATGCAGGGCCTGCACGCCATCCAGCTGCAGCTGGAGGAGGAGATGTT<br>CAACGGCGGCATCCGGAGATTTGAGGCAGACCAGCAGAGGCAGATC<br>GCCAGCGGCAACGAGTCCGATACCGCCTGGAATAGGCGCCTGCTGA<br>GCGAGCTGATCGCCCCAATGGCCGAGGGCATCCAGGCCTACAAGGA<br>GGAGTATGAGGGCAAGCGGGGCAGAGCACCAAGAGCCCTGGCCTTC<br>ATCAACTGCGTGGAGAATGAGGTGGCCGCCTACATCACCATGAAGAT<br>CGTGATGGACATGCTGAACACCGATGTGACACTGCAGGCCATCGCCA<br>TGAATGTGGCCGATAGGATCGAGGACCAGGTGCGCTTCTCCAAGCTG<br>GAGGGACACGCAGCCAAGTACTTTGAGAAGGTGAAGAAGTCTCTGA<br>AGGCCAGCAAGACCAAGTCCTATAGGCACGCACACAACGTGGCAGT<br>GGTGGCAGAGAAGTCTGTGGCCGACCGGGATGCCGACTTCAGCAGA<br>TGGGAGGCCTGGCCAAAGGACACCCTGCTGCAGATCGGCATGACACT<br>GCTGGAGATCCTGGAGAACAGCGTGTTCTTCAATGGCCAGCCCGTGT<br>TCCTGCGGACCCTGAGAACAAATGGCGGCAAGCACGGCGTGTACTAT<br>CTGCAGACCTCCGAGCACGTGGGCGAGTGGATCACAGCCTTTAAGGA

	GCACGTGGCACAGCTGTCTCCAGCATACGCACCTTGCGTGATCCCAC<br>CTAGGCCATGGGTGAGCCCCTTCAACGGAGGATTTCACACCGAGAAG<br>GTGGCCTCCAGGATCCGCCTGGTGAAGGGCAATAGGGAGCACGTGC<br>GCAAGCTGACCAAGAAGCAGATGCCCGAGGTGTACAAGGCCGTGAA<br>CGCCCTGCAGGCCACAAAGTGGCAAGTGAATAAGGAGGTGCTGCAG<br>GTGGTGGAGGACGTGATCAGGCTGGATCTGGGCTATGGCGTGCCTTC<br>CTTCAAGCCACTGATCGACCGCGAGAACAAGCCTGCCAATCCAGTGC<br>CCCTGGAGTTTCAGCACCTGCGGGGCAGAGAGCTGAAGGAGATGCT<br>GACCCCAGAGCAGTGGCAGGCCTTCATCAACTGGAAGGGCGAGTGT<br>ACAAAGCTGTACACCGCCGAGACAAAGAGGGGCTCTAAGAGCGCCG<br>CAACCGTGAGGATGGTGGGACAGGCCCGCAAGTACTCTCAGTTCGAC<br>GCCATCTACTTCGTGTACGCCCTGGATTCCAGGTCTCGCGTGTATGCC<br>CAGAGCTCCACCCTGTCTCCTCAGAGCAATGACCTGGGCAAGGCCCT<br>GCTGCGGTTCACAGAGGGCCAGAGACTGGATAGCGCCGAGGCCCTG<br>AAGTGGTTTCTGGTGAACGGCGCCAACAATTGGGGCTGGGACAAGA<br>AGACCTTCGATGTGCGGACAGCCAATGTGCTGGACAGCGAGTTTCAG<br>GATATGTGCAGAGACATCGCCGCCGATCCCCTGACCTTCACACAGTG<br>GGTGAACGCCGATTCCCCTTATGGCTTCCTGGCCTGGTGTTTTGAGTA<br>CGCCAGGTATCTGGATGCCCTGGACGAGGGCACCCAGGACCAGTTTA<br>TGACACACCTGCCAGTGCACCAGGATGGCAGCTGCTCCGGCATCCAG<br>CACTACAGCGCCATGCTGAGGGACGCAGTGGGAGCCAAGGCCGTGA<br>ATCTGAAGCCCTCTGACAGCCCTCAGGATATCTATGGCGCCGTGGCC<br>CAGGTGGTCATCCAGAAGAACTACGCCTATATGAATGCCGAGGACGC<br>CGAGACCTTCACATCCGGATCTGTGACCCTGACAGGAGCAGAGCTGC<br>GGTCCATGGCCTCTGCCTGGGATATGATCGGCATCACCAGAGGCCTG<br>ACAAAGAAGCCAGTGATGACCCTGCCATACGGAAGCACCAGGCTGA<br>CATGTAGAGAGTCCGTGATCGACTATATCGTGGATCTGGAGGAGAAG<br>GAGGCACAGAGGGCAATCGCAGAGGGAAGGACAGCAAACCCTGTGC<br>ACCCATTTGATAATGACAGGAAGGACTCTCTGACCCCAAGCGCCGCC<br>TACAACTATATGACAGCCCTGATCTGGCCTAGCATCTCCGAGGTGGT<br>GAAGGCCCCAATCGTGGCCATGAAGATGATCAGGCAGCTGGCCAGG<br>TTCGCAGCAAAGAGGAATGAGGGCCTGGAGTACACCCTGCCAACAG<br>GCTTTATCCTGCAGCAGAAGATCATGGCCACCGATATGCTGAGAGTG<br>TCCACATGCCTGATGGGCGAGATCAAGATGTCTCTGCAGATCGAGAC<br>CGATGTGGTGGACGAGACAGCAATGATGGGAGCTGCCGCCCCAAAC<br>TTCGTGCACGGACACGACGCCTCCCACCTGATCCTGACCGTGTGCGA<br>TCTGGTGGACAAGGGCATCACATCCATCGCCGTGATCCACGATTCTT<br>TTGGCACCCACGCAGGAAGAACAGCAGATCTGAGGGACTCTCTGAG<br>AGCCGAGATGGTGAAGATGTACCAGGGCAGGAACGCCCTGCAGAGC<br>CTGCTGGACGAGCACGAGGAGCGGTGGCTGGTGGATACCGGCATCC<br>AGGTGCCTGAGCAGGGCGAGTTCGACCTGAATGAGATCCTGGTGAGC<br>GATTATTGTTTTGCCTAA
K11 RNAP	ATGAACGCCCTGAACATCGCCCGGAACGACTTTAGCGAAATCGAGCT<br>GGCCGCAATCCCCTACAACATCCTGTCTGAACACTACGGCGACAAGC<br>TGGCCCGCGAGCAGCTGGCCCTGGAGCACGAGGCCTACGAGCTGGG<br>CGAGCAAAGATTCCTGAAGATGCTGGAAAGACAGGTGAAAGCCGGC<br>GAGTTCGCCGACAACGCCGCCGCCAAACCTCTGGTACTGACCCTGCA<br>CCCTCAGTTGACCAAGAGAATCGACGATTGGAAAGAGGAACAGGCT<br>AACGCCAGAGGCAAGAAGCCTAGAGCCTATTACCCTATCAAGCACG<br>GCGTGGCCTCTAAGCTCGCAGTGTCCATGGGCGCCGAAGTGCTGAAG<br>GAGAAGCGGGGCGTGTCCAGCGAGGCAATCGCGCTGCTGACCATTA<br>AGGTGGTGCTGGGCACCCTGACCGATGCCTCTAAGGCCACAATCCAG<br>CAAGTGAGCAGCCAGCTGGGCAAGGCTCTGGAGGACGAGGCCAGAT<br>TCGGCAGAATCAGAGAGCAGGAGGCAGCATACTTCAAGAAGAACGT<br>GGCCGACCAACTGGACAAGAGAGTGGGCCACGTGTACAAGAAGGCC<br>TTCATGCAGGTGGTCGAGGCCGACATGATCAGCAAGGGCATGCTAGG<br>AGGCGATAATTGGGCCAGCTGGAAGACAGATGAACAGATGCACGTT

	GGCACCAAGCTGCTGGAACTGCTGATTGAGGGCACAGGCCTGGTCGA<br>GATGACCAAGAACAAGATGGCCGACGGCAGCGACGACGTGACGAGC<br>ATGCAGATGGTGCAGCTGGCCCCTGCCTTTGTCGAACTGCTGAGCAA<br>GCGGGCCGGTGCCCTGGCTGGAATCAGCCCCATGCACCAGCCTTGTG<br>TGGTGCCCCCCAAGCCCTGGGTGGAAACAGTGGGAGGCGGCTACTG<br>GTCCGTGGGACGCAGACCCCTGGCCCTGGTGCGGACCCACAGCAAA<br>AAAGCCCTCCGGCGGTACGCCGACGTGCACATGCCTGAAGTTTACAA<br>GGCCGTGAACCTGGCCCAAAACACTCCATGGAAGGTGAACAAGAAG<br>GTCCTGGCGGTGGTGAATGAGATCGTGAACTGGAAGCACTGTCCTGT<br>GGGAGATGTGCCTGCTATCGAGCGGGAAGAGCTGCCTCCAAGACCTG<br>ACGACATCGATACAAACGAAGTGGCCAGAAAGGCTTGGCGAAAAGA<br>GGCCGCTGCCGTGTATAGAAAGGACAAGGCCCGGCAGAGCAGAAGA<br>CTGAGCATGGAGTTCATGGTGGCCCAAGCCAACAAATTCGCCAATCA<br>CAAGGCCATCTGGTTCCCTTATAACATGGACTGGCGGGGCAGAGTGT<br>ACGCCGTGTCTATGTTCAACCCCCAGGGCAACGACATGACAAAGGGC<br>ATGCTGACCCTGGCCAAGGGCAAGCCCATCGGCCTGGATGGCTTTTA<br>CTGGCTGAAGATCCACGGCGCCAACTGCGCTGGCGTGGACAAGGTTC<br>CTTTTCCAGAGAGAATCAAGTTCATCGAAGAAAACGAGGGCAACATC<br>CTGGCCTCCGCCGCCGATCCTCTGAACAACACCTGGTGGACCCAGCA<br>GGACAGCCCTTTTTGCTTTCTGGCTTTCTGCTTCGAGTACGCCGGAGT<br>GAAACACCACGGACTGAACTACAACTGTAGCCTGCCTCTGGCTTTCG<br>ACGGCAGCTGTAGCGGCATCCAGCACTTCAGCGCAATGCTGAGGGAC<br>TCTATCGGAGGAAGAGCCGTGAACCTGCTGCCTAGCGACACCGTGCA<br>GGACATCTACAAAATCGTGGCCGACAAGGTGAACGAGGTGCTGCAC<br>CAGCACGCCGTCAATGGCAGCCAAACAGTGGTGGAGCAGATCGCCG<br>ACAAGGAAACAGGCGAGTTCCATGAAAAGGTGACACTGGGCGAATC<br>CGTGCTGGCTGCCCAGTGGCTGCAATACGGTGTCACAAGAAAGGTGA<br>CCAAGCGGAGCGTGATGACACTCGCTTACGGCTCTAAGGAGTTTGGT<br>TTCAGACAGCAAGTGCTGGAAGATACCATCCAGCCAGCCATTGACAA<br>TGGCGAAGGACTGATGTTCACCCACCCCAACCAGGCGGCTGGATACA<br>TGGCCAAGCTGATCTGGGACGCCGTGACCGTGACCGTGGTGGCCGCT<br>GTTGAGGCCATGAACTGGCTGAAAAGCGCCGCCAAGCTGCTGGCTGC<br>TGAGGTGAAGGACAAAAAGACCAAAGAGGTGCTTAGAAAGCGGTGC<br>GCCATCCACTGGGTGACACCTGATGGATTTCCCGTGTGGCAGGAGTA<br>CAGAAAGCAGAACCAGGCCCGGCTGAAGCTGGTTTTCCTGGGGCAG<br>GCTAACGTGAAGATGACCTACAATACCGGCAAAGATTCTGAAATCGA<br>CGCCCACAAACAGGAGAGCGGCATAGCCCCTAATTTTGTGCATAGCC<br>AGGATGGCTCCCACCTGAGAATGACCGTGGTACATGCTAATGAGGTG<br>TACGGCATCGACAGCTTCGCTCTGATCCACGACAGCTTCGGCACCAT<br>CCCTGCCGACGCCGGCAATCTGTTCAAGGCCGTCAGAGAAACCATGG<br>TCAAGACCTATGAAGATAATGACGTGATCGCCGACTTCTACGACCAG<br>TTCGCCGATCAGCTGCATGAAAGCCAGCTGGACAAAATGCCTGCCGT<br>GCCCGCCAAGGGCGACCTGAACCTCCGAGATATCCTGGAGTCTGATT<br>TCGCCTTCGCCTGA
Phi15<br>RNAP	ATGATCGAGGTGGCTAAAAACGACTTCAGCGACGTGAAGACCGATT<br>GGGCCTTCCGGGTGTTGTCTGAACTGTACGGCGAAGAGCTGGCCGCC<br>GCTCAGCTGGCTCTGGAGCACGAGTCCCACGAAATCGGCGAGGCTAA<br>ATTCAAGAAGGCCCTGGACCGCCAAATGAAGCGGGGCGAGACAAGC<br>GAAACCAGCGTGGCTAAGCCCCTGGTGGCCATGCTCGTCCCAAAATT<br>TGTGGAAAAGATGGACGCCTGGGTGGAACACCAGATGAAGAACGTA<br>CGGAGAAAGTCTGTGGCCCTGAAGTTCATCCAGATGGTGGCTACAGA<br>GAGAGTCGCCGTGATCACCATCAAAACCGTGATCAACGCCATGAGCC<br>AGGGCGACGTGGTGCTGCAAGCCATCGCCGGAAGAATCGGCCGAGG<br>CATCGAAGAAGAGGCTAGATTCGGCAGGATTCGGGATCAGGAAGCT<br>AAGCACTTCAAGAAATACATCAGAGAAGCCCTTAACAAACGGAACG<br>GCCACACTTACAAGAGAGCCTACATGCACGCCGTGGAAGATAGAAT<br>GCTGGAAGCTGGCGAACTGAATGGCGCCTGGTCCGATTGGGACAAC

	GAGGATCCTACCATCATCGCCCACATCGGACTCAGATGCATCGAGGC GCTGATTGAGAGCTCTGGCCTGGTTAGAATCACAAGAAGAAGCGCCG GAAACGTGAAAGAGGACTGCAACGTGCTGGAGCTGGAGCCTCAGTG GGTTGAAATGCTGAACCAGAGGGCCTTCACCCTGGCCGGCGTGAACA CATACCACCAGCCTTGTGTGGTGCCCCCCAGACCCTGGACAAGACCT GTGGGCGGAGGCTACTGGGGAAAGGGAAGACGGCCTACCCGGTTCA TCAGAGTGCACAACAAGAAGGCTCTGGAGCGGTACAGAGATGTCGA CATGGAAGCCGTGTATAAGGCCGTGAACATCGCCCAGAACACCGCCT GGAGCATCAACAAGAGAATACTGGAAGTGGCCGAGGCCCTGGCCTC TTGGACCAACGTGCCAATCTCTAAGTGGCCTAAGGCCGAGACGCAGG AACTGCCTGTGAAGCCCCACGACATCGAAACCAATGAGGAAGCCCG GAACGCCTGGAAGAAACAGGCCAGCGGAGTGTACCGGAGCGAAAGC AGCAGAGTGAGCAGAAGAATGAGTCTGGAAACCACCCTGGAAACCG CCCGGAAGTTCGCCGACTTCGAGGCCATCTACTTCCCCCACAATCTG GACTGGCGGGGCAGAGTGTACGCCCTACCCGTCTTTAACCCTCAGAG AGATGATCTGACCAAGGGCCTTCTTCAAGCCAGCAAGGGCGAGCCTG TCGGCGAGGACGGAATCAAGTGGCTGATGATCCACGGCGCCAACAC CGCCGGAGTTGATAAGGTGCCCTTTGACGAAAGGCAGCAGTGGGTGC GCGACAATGAAAGAACAATCCTGCAATGTGCCGAAGACCCTCTGACC CACACAGAGTGGATGAGCATGGATAGCCCTTTCTGCTTCCTGGCATT TTGCTTTGAGTGGGCCGGCGTGGTGAAGGACGGCCCTAATCACGTGT CCGCCCTGCCTATCGCCTTTGACGGCAGCTGTAGCGGCATTCAGCAC TTCAGCGCCATGCTGAGAGATGAGACAGGAGGAAGAGCCGTGAACC TGCTGCCCAGCGAGCGCGTGCAGGATATCTACAGACTGGTCAGCGAC GGCGTGAATGCCGCTCTGAGAGACGATGCCGTGCACGGCACCGACG ACAGCACAGACGTGCACGTGGACGAGAAGACCGGCGAGATCACAGA AAGAAGAGTGCTGGGCACTAGAACCCTCGCCGCGGCTTGGCTGGCAC ATGGCGTGGACCGGAGCGTGACCAAGCGGAGCGTTATGACCCTGGC CTATGGCAGCAAGGAGTTCGGCTTCACCGACCAAGTGCGGGATGACA TTATCACACCTGCCGTGGATGCCGGCAGCCTGAACTTCCCTCAGCCA CAACAGGCCGCCCGCTACATGGCCCATCTGATCTGGGTGAGCGTGGG CAAGACAGTGGTGGCGGCCGTCGAGGCTATGGAATGGCTGCAAAAG AGCGCGAAACTGCTGGCTGCAATCGTGAAGGAAAAGAAGGGCCCTG AGAAGGGCAAGATTCTGAAGCCTGCTATGCCAGTGTACTGGGTCACC CCTGATGGTTTTCCGGTGTGGCAGGAGTACCGGGTGCAGCAGGCCAA AAGAATCGACATGATCCTGATGGGCGACGTCAGACTGACCGCCACA GTGCTGCACCAGCAGGACCAAATCGACGCTAGAAAGCAGGAGTCTG GCATCTCCCCGAATTTCGTGCATTCAATGGACGGCAACCACCTGAGA CAGACCGTGGTGCATGCCCACGACGCCTACGACATCACCTTCTTCGC CCTGATCCACGATAGCTTCGGGACAATCCCTGCCAAGGCCGGCCAGC TGTTCAAGGCGGTGCGGGAAACCATGGTGACCGCCTACGAGCACAA CGACGTGCTGGCCGACTTCAGAGAGCAGTTCATCGACCAGCTGCACG AGACACAGATGGACAAGATGCCTGAACTGCCTAAGAAGGGCACCCT GGATATCAGAGAGATCCTGAAAAGCCAGTTTGCCTTCGCCTGA
Hemagglutinin (HA/Brisbane, H1N1, GenBank Sequence ID: ACA28844.1, Codon optimized)	ATGAAGGTGAAACTGCTGGTGCTGCTGTGTACCTTCACCGCCACCTA CGCCGACACCATCTGCATCGGCTACCACGCGAATAACAGCACCGACA CCGTGGACACCGTGCTGGAGAAGAACGTGACCGTGACCCACAGCGT GAACCTGCTTGAAAACTCCCACAACGGCAAGCTGTGCCTGCTGAAGG GCATCGCCCCCCTGCAGCTGGGCAACTGCAGCGTGGCCGGCTGGATC CTGGGCAACCCCGAGTGCGAGCTGCTGATCAGCAAGGAGAGCTGGA GCTACATCGTGGAGAAGCCCAACCCCGAGAACGGAACATGCTACCC CGGCCACTTCGCCGACTACGAGGAGCTGAGAGAGCAGCTGAGCAGC GTGAGCAGCTTCGAGAGATTCGAAATCTTCCCCAAGGAGAGCAGCTG GCCCAACCACACCGTGACCGGCGTGAGCGCCAGCTGCAGCCACAAT GGCGAAAGCAGCTTCTACAGAAACCTGCTGTGGCTGACCGGCAAGA ACGGCCTGTACCCCAACCTGAGCAAGAGCTACGCCAACAACAAAGA GAAGGAGGTGCTGGTGCTGTGGGGCGTGCACCACCCCCCCAACATCG

	GCGACCAGAAGGCCCTGTACCACACCGAGAACGCCTACGTGAGCGT<br>GGTGAGCAGCCACTACAGCAGAAAGTTCACCCCCGAGATCGCCAAG<br>AGACCCAAGGTGAGAGACCAGGAGGGCAGAATCAACTACTACTGGA<br>CCCTGCTGGAGCCCGGCGACACCATCATCTTCGAGGCCAACGGCAAC<br>CTGATCGCCCCCAGATACGCCTTCGCCCTGAGCAGAGGCTTCGGCAG<br>CGGCATCATCAACAGCAACGCCCCCATGGACAAGTGCGACGCCAAG<br>TGCCAGACCCCCCAGGGCGCCATCAACAGCAGCCTGCCCTTCCAGAA<br>CGTGCACCCCGTGACCATCGGCGAGTGCCCCAAGTACGTGAGAAGCG<br>CCAAGCTGAGAATGGTGACCGGCCTGAGAAACATCCCCAGCATCCA<br>GAGCAGAGGCCTGTTCGGCGCCATCGCCGGCTTTATCGAGGGCGGCT<br>GGACCGGCATGGTGGACGGCTGGTACGGCTACCACCACCAGAACGA<br>GCAGGGCAGCGGCTACGCCGCCGACCAAAAGAGCACCCAGAACGCC<br>ATCAACGGCATCACCAACAAGGTGAACAGCGTGATCGAGAAGATGA<br>ACACCCAGTTCACCGCCGTGGGCAAGGAGTTCAACAAGCTGGAGAG<br>AAGAATGGAGAACCTGAACAAGAAGGTGGACGACGGCTTCATCGAT<br>ATTTGGACCTACAACGCCGAGCTGTTGGTCCTCTTGGAGAATGAAAG<br>AACCCTGGACTTCCACGACTCCAACGTGAAAAATCTGTACGAGAAGG<br>TCAAGAGCCAGCTGAAGAACAACGCCAAGGAGATCGGCAACGGCTG<br>CTTCGAGTTCTACCACAAATGTAACGACGAGTGCATGGAGAGCGTGA<br>AGAACGGCACGTACGACTACCCCAAGTACAGCGAGGAGAGCAAGCT<br>GAACAGAGAGAAGATCGACGGCGTGAAGCTGGAGAGCATGGGCGTG<br>TACCAGATCCTGGCCATCTACAGCACCGTGGCCAGCAGCTTGGTACT<br>GCTGGTGTCACTGGGCGCCATCAGCTTCTGGATGTGCAGCAACGGCA<br>GCCTGCAGTGCAGAATCTGCATCTAA
Neuraminidase (NA/WSN, H1N1, GenBank Sequence ID: ACF54601.1, Codon optimized)	ATGAACCCCAATCAGAAGATCATCACCATCGGCAGCATCTGCATGGT<br>GGTGGGCATCATCAGCCTGATCCTGCAGATCGGCAACATCATCAGCA<br>TCTGGATCAGCCACAGCATTCAGACCGGCAATCAGAACCACACCGGC<br>ATCTGCAACCAAGGCAGCATCACCTACAAGGTGGTGGCCGGCCAAG<br>ACAGCACAAGCGTGATCCTGACCGGCAACAGCAGCCTGTGCCCCATC<br>AGAGGCTGGGCCATCCACAGCAAGGATAACGGCATCCGAATCGGCA<br>GCAAGGGCGACGTGTTCGTGATCAGAGAGCCCTTCATCAGCTGCAGC<br>CACCTGGAGTGCAGAACCTTCTTCCTGACCCAAGGCGCCCTGCTGAA<br>CGACAAGCACAGCAGAGGCACCTTCAAGGACAGAAGCCCCTACAGA<br>GCCCTGATGAGCTGCCCCGTGGGCGAGGCCCCTAGCCCCTACAACAG<br>CAGATTCGAGAGCGTGGCCTGGAGCGCTAGCGCCTGCCATGACGGCA<br>TGGGCTGGCTGACCATCGGCATCTCCGGCCCGGACGACGGCGCCGTG<br>GCCGTGCTGAAGTATAATGGTATCATAACCGAGACCATCAAGAGCTG<br>GAGAAAGAACATCCTGAGAACCCAAGAGAGCGAGTGCACCTGCGTG<br>AACGGCAGCTGCTTCACCATCATGACCGACGGCCCTAGCGACGGCCT<br>GGCTAGCTACAAAATCTTCAAGATCGAGAAGGGCAAGGTGACCAAG<br>AGCATCGAGCTGAACGCCCCCAACAGCCACTACGAGGAGTGCAGCT<br>GCTACCCCGACACCGGCAAGGTGATGTGCGTGTGCAGAGACAACTG<br>GCACGGCAGCAACAGACCCTGGGTGAGCTTCGATCAGAACCTGGACT<br>ACAAGATCGGCTACATCTGCAGCGGCGTGTTTGGGGACAACCCTAGA<br>CCTAAAGACGGCACCGGAAGCTGTGGACCCGTGAGCGCCGACGGCG<br>CCAACGGCGTGAAGGGCTTCAGCTACAAGTACGGCAACGGCGTGTG<br>GATCGGCAGAACCAAGAGCGACAGCAGCAGACACGGCTTCGAGATG<br>ATCTGGGACCCCAACGGCTGGACCGAGACCGACAGCAGATTCAGCA<br>TGAGACAAGACGTGGTGGCCATGACCGACAGAAGCGGCTACAGCGG<br>CAGCTTCGTGCAGCACCCCGAGCTGACCGGCCTGGACTGCATGAGAC<br>CCTGCTTCTGGGTGGAGCTGATCAGAGGCCTGCCCGAGGAGAACGCC<br>ATCTGGACAAGCGGCAGCATCATCAGCTTCTGCGGCGTGAACAGCGA<br>CACCGTGGACTGGAGCTGGCCCGACGGCGCCGAGCTGCCCTTCACCA<br>TCGACAAGTAA
Matrix protein 1 (M1/Puerto,	ATGAGCCTGCTGACCGAGGTGGAGACCTACGTGCTGAGCATCATCCC<br>CAGCGGCCCCCTGAAGGCCGAGATCGCCCAGAGACTGGAGGACGTG<br>TTCGCCGGCAAGAACACCGACCTGGAGGTGCTGATGGAGTGGCTGA

H1N1, GenBank Sequence ID: NP_040978 .1, Codon optimized)	AGACCAGACCCATCCTGAGCCCCCTGACCAAGGGCATCCTGGGCTTC<br>GTGTTCACCCTGACCGTGCCCAGCGAGAGAGGCCTGCAGAGAAGAA<br>GATTCGTGCAGAACGCCCTGAACGGCAACGGCGACCCCAACAACAT<br>GGACAAGGCCGTGAAGCTGTACAGAAAGCTGAAGAGAGAGATCACC<br>TTCCACGGCGCCAAGGAGATCAGCCTGAGCTACAGCGCCGGCGCCCT<br>GGCCAGCTGCATGGGCCTGATCTACAACAGAATGGGCGCCGTGACCA<br>CCGAGGTGGCCTTCGGCCTGGTGTGCGCCACCTGCGAGCAGATCGCC<br>GACAGCCAGCACAGAAGCCACAGGCAGATGGTGACCACCACCAACC<br>CCCTGATCAGACACGAGAACAGAATGGTGCTGGCCAGCACGACCGC<br>CAAGGCCATGGAGCAGATGGCCGGCTCCAGCGAACAGGCGGCTGAG<br>GCCATGGAGGTGGCCAGCCAGGCCCGACAGATGGTGCAGGCCATGA<br>GAACCATCGGCACCCACCCCAGCAGCAGCGCCGGCCTGAAGAACGA<br>CCTGCTGGAGAACCTGCAGGCCTACCAGAAGAGAATGGGCGTGCAG<br>ATGCAGAGATTCAAGTAA
GFP-M1	ATGGTGAGCAAGGGCGAGGAGCTGTTCACCGGGGTGGTGCCCATCCT<br>GGTCGAGCTGGACGGCGACGTAAACGGCCACAAGTTCAGCGTGTCC<br>GGCGAGGGCGAGGGCGATGCCACCTACGGCAAGCTGACCCTGAAGT<br>TCATCTGCACCACCGGCAAGCTGCCCGTGCCCTGGCCCACCCTCGTG<br>ACCACCCTGACCTACGGCGTGCAGTGCTTCAGCCGCTACCCCGACCA<br>CATGAAGCAGCACGACTTCTTCAAGTCCGCCATGCCCGAAGGCTACG<br>TCCAGGAGCGCACCATCTTCTTCAAGGACGACGGCAACTACAAGACC<br>CGCGCCGAGGTGAAGTTCGAGGGCGACACCCTGGTGAACCGCATCG<br>AGCTGAAGGGCATCGACTTCAAGGAGGACGGCAACATCCTGGGGCA<br>CAAGCTGGAGTACAACTACAACAGCCACAACGTCTATATCATGGCCG<br>ACAAGCAGAAGAACGGCATCAAGGTGAACTTCAAGATCCGCCACAA<br>CATCGAGGACGGCAGCGTGCAGCTCGCCGACCACTACCAGCAGAAC<br>ACCCCCATCGGCGACGGCCCCGTGCTGCTGCCCGACAACCACTACCT<br>GAGCACCCAGTCCGCCCTGAGCAAAGACCCCAACGAGAAGCGCGAT<br>CACATGGTCCTGCTGGAGTTCGTGACCGCCGCCGGGATCACTCTCGG<br>CATGGACGAGCTGTACAAGTCCGGACTCAGATCTATGAGCCTGCTGA<br>CCGAGGTGGAGACCTACGTGCTGAGCATCATCCCCAGCGGCCCCCTG<br>AAGGCCGAGATCGCCCAGAGACTGGAGGACGTGTTCGCCGGCAAGA<br>ACACCGACCTGGAGGTGCTGATGGAGTGGCTGAAGACCAGACCCAT<br>CCTGAGCCCCCTGACCAAGGGCATCCTGGGCTTCGTGTTCACCCTGA<br>CCGTGCCCAGCGAGAGAGGCCTGCAGAGAAGAAGATTCGTGCAGAA<br>CGCCCTGAACGGCAACGGCGACCCCAACAACATGGACAAGGCCGTG<br>AAGCTGTACAGAAAGCTGAAGAGAGAGATCACCTTCCACGGCGCCA<br>AGGAGATCAGCCTGAGCTACAGCGCCGGCGCCCTGGCCAGCTGCATG<br>GGCCTGATCTACAACAGAATGGGCGCCGTGACCACCGAGGTGGCCTT<br>CGGCCTGGTGTGCGCCACCTGCGAGCAGATCGCCGACAGCCAGCACA<br>GAAGCCACAGGCAGATGGTGACCACCACCAACCCCCTGATCAGACA<br>CGAGAACAGAATGGTGCTGGCCAGCACGACCGCCAAGGCCATGGAG<br>CAGATGGCCGGCTCCAGCGAACAGGCGGCTGAGGCCATGGAGGTGG<br>CCAGCCAGGCCCGACAGATGGTGCAGGCCATGAGAACCATCGGCAC<br>CCACCCCAGCAGCAGCGCCGGCCTGAAGAACGACCTGCTGGAGAAC<br>CTGCAGGCCTACCAGAAGAGAATGGGCGTGCAGATGCAGAGATTCA<br>AGTAA
NA(1-61aa)-mCherry	ATGAACCCCAATCAGAAGATCATCACCATCGGCAGCATCTGCATGGT<br>GGTGGGCATCATCAGCCTGATCCTGCAGATCGGCAACATCATCAGCA<br>TCTGGATCAGCCACAGCATTCAGACCGGCAATCAGAACCACACCGGC<br>ATCTGCAACCAAGGCAGCATCACCTACAAGGTGGTGGCCGGCagatctA<br>TGGTGAGCAAGGGCGAGGAGGATAACATGGCCATCATCAAGGAGTT<br>CATGCGCTTCAAGGTGCACATGGAGGGCTCCGTGAACGGCCACGAGT<br>TCGAGATCGAGGGCGAGGGCGAGGGCCGCCCCTACGAGGGCACCCA<br>GACCGCCAAGCTGAAGGTGACCAAGGGTGGCCCCCTGCCCTTCGCCT<br>GGGACATCCTGTCCCCTCAGTTCATGTACGGCTCCAAGGCCTACGTG<br>AAGCACCCCGCCGACATCCCCGACTACTTGAAGCTGTCCTTCCCCGA

	GGGCTTCAAGTGGGAGCGCGTGATGAACTTCGAGGACGGCGGCGTG GTGACCGTGACCCAGGACTCCTCCCTGCAGGACGGCGAGTTCATCTA CAAGGTGAAGCTGCGCGGCACCAACTTCCCCTCCGACGGCCCCGTAA TGCAGAAGAAGACCATGGGCTGGGAGGCCTCCTCCGAGCGGATGTA CCCCGAGGACGGCGCCCTGAAGGGCGAGATCAAGCAGAGGCTGAAG CTGAAGGACGGCGGCCACTACGACGCTGAGGTCAAGACCACCTACA AGGCCAAGAAGCCCGTGCAGCTGCCCGGCGCCTACAACGTCAACATC AAGTTGGACATCACCTCCCACAACGAGGACTACACCATCGTGGAACA GTACGAACGCGCCGAGGGCCGCCACTCCACCGGCGGCATGGACGAG CTGTACAAGTAA

Supplementary Table S10. Primers for Golden gate assembly.

Two-reporters Golden gate assembly primers	
position 1 primer F	5'-ATAGCTCTTCAGTCggaaaaggcctccacggccTAATACGACTCACTATAGGGGctcgag-3'
position 1 primer R	5'-ATAGCTCTTCATGCccgcgacgcgtCAAAAAACCCCTC-3'
position 2 primer F	5'-ATAGCTCTTCAGCAggaaaaggcctccacggccTAATACGACTCACTATAGGGGctcgag-3'
position 2 primer R	5'-ATAGCTCTTCACCGccgcgacgcgtCAAAAAACCCCTC-3'

Three-reporters/VLP Golden gate assembly primers	
position 1 primer F	5'-ATAGCTCTTCAGTCggaaaaggcctccacggccTAATACGACTCACTATAGGGGctcgag-3'
position 1 primer R	5'-ATAGCTCTTCATGCccgcgacgcgtCAAAAAACCCCTC-3'
position 2 primer F	5'-ATAGCTCTTCAGCAggaaaaggcctccacggccTAATACGACTCACTATAGGGGctcgag-3'
position 2 primer R	5'-ATAGCTCTTCACGTccgcgacgcgtCAAAAAACCCCTC-3'
position 3 primer F	5'-ATAGCTCTTCAACGggaaaaggcctccacggccTAATACGACTCACTATAGGGGctcgag-3'
position 3 primer R	5'-ATAGCTCTTCACCGccgcgacgcgtCAAAAAACCCCTC-3'
