# Supplementary material for: Precise programming of multigene expression stoichiometry in mammalian cells by a modular and programmable transcriptional system
Source: Nat Commun. 2023 Mar 17;14:1500. doi: 10.1038/s41467-023-37244-y (PMC10023750; doi:10.1038/s41467-023-37244-y)
Supplement: Supplementary file 2 — Reporting Summary [file 41467_2023_37244_MOESM2_ESM.pdf]

nature portfolio

Corresponding author(s): Long Qian and Chunbo Lou

Last updated by author(s): Mar 8,2023

Reporting Summary

Nature Portfolio wishes to improve the reproducibility of the work that we publish. This form provides structure for consistency and transparency in reporting. For further information on Nature Portfolio policies, see our Editorial Policies and the Editorial Policy Checklist.

Statistics

For all statistical analyses, confirm that the following items are present in the figure legend, table legend, main text, or Methods section.

n/a	Confirmed	
☐	☒	The exact sample size (*n*) for each experimental group/condition, given as a discrete number and unit of measurement
☐	☒	A statement on whether measurements were taken from distinct samples or whether the same sample was measured repeatedly
☒	☐	The statistical test(s) used AND whether they are one- or two-sided<br>*Only common tests should be described solely by name; describe more complex techniques in the Methods section.*
☒	☐	A description of all covariates tested
☐	☒	A description of any assumptions or corrections, such as tests of normality and adjustment for multiple comparisons
☐	☒	A full description of the statistical parameters including central tendency (e.g. means) or other basic estimates (e.g. regression coefficient) AND variation (e.g. standard deviation) or associated estimates of uncertainty (e.g. confidence intervals)
☒	☐	For null hypothesis testing, the test statistic (e.g. *F*, *t*, *r*) with confidence intervals, effect sizes, degrees of freedom and *P* value noted<br>*Give P values as exact values whenever suitable.*
☒	☐	For Bayesian analysis, information on the choice of priors and Markov chain Monte Carlo settings
☒	☐	For hierarchical and complex designs, identification of the appropriate level for tests and full reporting of outcomes
☒	☐	Estimates of effect sizes (e.g. Cohen's *d*, Pearson's *r*), indicating how they were calculated

Our web collection on statistics for biologists contains articles on many of the points above.

Software and code

Policy information about availability of computer code

Data collection	Transmission Electron Microscope (FEI Tecnai G2 F20) was used to collect images of influenza VLPs;Nikon Ti2-E microscope was used to observe co-localization events;Beckman Coulter CytoFlex S or BD FACSDiva Software version 8.0.2 (BD Biosciences) was used to collect all the Flow Cyto. data;Fluorescent VLP quantified by Apogee Micro Flow Cytometer.
Data analysis	Public and commercial software: Matlab R2016 (Mathworks); Python 3.7.3; Imaris software 8.3(Oxford Instruments);CytExpert Version 2.4.0.28; Histogram software version 6.0.29(Apogee Flow Systems).<br>Custom Matlab codes were used to acquire parameters in two-reporter system and make predictions in three-reporter system. Custom python codes were used to acquire parameters in VLP production and make predictions(codes are available from the corresponding author on request).

For manuscripts utilizing custom algorithms or software that are central to the research but not yet described in published literature, software must be made available to editors and reviewers. We strongly encourage code deposition in a community repository (e.g. GitHub). See the Nature Portfolio guidelines for submitting code & software for further information.

Data

Policy information about availability of data

All manuscripts must include a data availability statement. This statement should provide the following information, where applicable:

- Accession codes, unique identifiers, or web links for publicly available datasets
- A description of any restrictions on data availability
- For clinical datasets or third party data, please ensure that the statement adheres to our policy

Source data are provided with this paper. All other data are available from the corresponding author on request. Representative plasmid sequences have been deposited to website. (http://bdainformatics.org/dataRepository)

Human research participants

Policy information about studies involving human research participants and Sex and Gender in Research.

Reporting on sex and gender	*Use the terms sex (biological attribute) and gender (shaped by social and cultural circumstances) carefully in order to avoid confusing both terms. Indicate if findings apply to only one sex or gender; describe whether sex and gender were considered in study design whether sex and/or gender was determined based on self-reporting or assigned and methods used. Provide in the source data disaggregated sex and gender data where this information has been collected, and consent has been obtained for sharing of individual-level data; provide overall numbers in this Reporting Summary. Please state if this information has not been collected. Report sex- and gender-based analyses where performed, justify reasons for lack of sex- and gender-based analysis.*
Population characteristics	*Describe the covariate-relevant population characteristics of the human research participants (e.g. age, genotypic information, past and current diagnosis and treatment categories). If you filled out the behavioural & social sciences study design questions and have nothing to add here, write "See above."*
Recruitment	*Describe how participants were recruited. Outline any potential self-selection bias or other biases that may be present and how these are likely to impact results.*
Ethics oversight	*Identify the organization(s) that approved the study protocol.*

Note that full information on the approval of the study protocol must also be provided in the manuscript.

Field-specific reporting

Please select the one below that is the best fit for your research. If you are not sure, read the appropriate sections before making your selection.

☒ Life sciences ☐ Behavioural & social sciences ☐ Ecological, evolutionary & environmental sciences

For a reference copy of the document with all sections, see nature.com/documents/nr-reporting-summary-flat.pdf

Life sciences study design

All studies must disclose on these points even when the disclosure is negative.

Sample size	For all experiments in the study, sample sizes were chosen to align with what is typical in the field.
Data exclusions	No data were excluded intentionally from the analysis.
Replication	Experiments were repeated at least twice to confirm the reproducibility.
Randomization	As we performed experiments using cell lines with defined genetic background, there were no need for randomization.
Blinding	Investigators were not blinded.We always set control and repeat experiment group for this study.

Reporting for specific materials, systems and methods

We require information from authors about some types of materials, experimental systems and methods used in many studies. Here, indicate whether each material, system or method listed is relevant to your study. If you are not sure if a list item applies to your research, read the appropriate section before selecting a response.

Materials & experimental systems

n/a	Involved in the study
☒	☐ Antibodies
☐	☒ Eukaryotic cell lines
☒	☐ Palaeontology and archaeology
☒	☐ Animals and other organisms
☒	☐ Clinical data
☒	☐ Dual use research of concern

Methods

n/a	Involved in the study
☒	☐ ChIP-seq
☐	☒ Flow cytometry
☒	☐ MRI-based neuroimaging

Eukaryotic cell lines

Policy information about cell lines and Sex and Gender in Research

Cell line source(s)	Human Embryonic Kidney 293T cells (HEK293T, ATCC); Chinese Hamster Ovary cells (CHO, National Infrastructure Cell Line Resource, NICR). The generation of capping-T7 RNAP stable cell line was based on transgene integration in Chinese Hamster Ovary cells.
Authentication	The cell lines we used were authenticated by comparing the cell morphology and doubling time with data from the provider.
Mycoplasma contamination	All cell lines were tested negative for mycoplasma contamination.
Commonly misidentified lines (See ICLAC register)	We affirm that there are no commonly misidentified cell lines.

Flow Cytometry

Plots

Confirm that:

☒ The axis labels state the marker and fluorochrome used (e.g. CD4-FITC).

☒ The axis scales are clearly visible. Include numbers along axes only for bottom left plot of group (a 'group' is an analysis of identical markers).

☒ All plots are contour plots with outliers or pseudocolor plots.

☒ A numerical value for number of cells or percentage (with statistics) is provided.

Methodology

Sample preparation	Cells were cultured in plastic-bottom wells and dispersed by trypsin. Then, cells were suspended and transferred to 96 well plates.
Instrument	Fluorescence signals of all samples were collected on a Beckman Coulter CytoFlex S flow cytometry or BD Fortessa SORP equipped with proper lasers and filters.
Software	CytExpert Version 2.4.0.28 was used in this study to analyze the fluorescence signals.
Cell population abundance	In each flow cytometry assays in this study, at least 100,000 cells were collected.
Gating strategy	For all flow cytometry sample, we included a transfection marker and non-transfected cells (blank) to define the boundaries of positive and negative populations.

☒ Tick this box to confirm that a figure exemplifying the gating strategy is provided in the Supplementary Information.
